# GRANA: An AI–based tool for accelerating chloroplast grana nanomorphology analysis using hybrid intelligence

**DOI:** 10.1093/plphys/kiaf212

**Published:** 2025-05-23

**Authors:** Alicja Bukat, Marek Bukowicki, Michał Bykowski, Karolina Kuczkowska, Szymon Nowakowski, Anna Śliwińska, Łucja Kowalewska

**Affiliations:** Department of Plant Anatomy and Cytology, Faculty of Biology, University of Warsaw, Miecznikowa 1, 02-096 Warsaw, Poland; Center for Machine Learning, Faculty of Physics, University of Warsaw, Pasteura 5, 02-093 Warsaw, Poland; Centre of New Technologies, University of Warsaw, Banacha 2c, 02-093 Warsaw, Poland; Department of Plant Anatomy and Cytology, Faculty of Biology, University of Warsaw, Miecznikowa 1, 02-096 Warsaw, Poland; Department of Ecology and Environmental Conservation, Faculty of Biology, University of Warsaw, Miecznikowa 1, 02-096 Warsaw, Poland; Center for Machine Learning, Faculty of Physics, University of Warsaw, Pasteura 5, 02-093 Warsaw, Poland; Faculty of Mathematics, Informatics and Mechanics, Institute of Applied Mathematics, University of Warsaw, Banacha 2, 02-097 Warsaw, Poland; Center for Machine Learning, Faculty of Physics, University of Warsaw, Pasteura 5, 02-093 Warsaw, Poland; Department of Plant Anatomy and Cytology, Faculty of Biology, University of Warsaw, Miecznikowa 1, 02-096 Warsaw, Poland

## Abstract

Grana are fundamental structural units of the intricate chloroplast membrane network. Investigating their nanomorphology is essential for understanding photosynthetic efficiency regulation. Here, we present GRANA (Graphical Recognition and Analysis of Nanostructural Assemblies), an artificial intelligence–enhanced, user-friendly software tool that recognizes grana on thylakoid network electron micrographs and generates a complex set of their structural parameters. GRANA employs 3 artificial neural networks of different architectures and binds them in a 1-click workflow. Its output is designed to facilitate hybrid intelligence analysis, securing fast and reliable results from large datasets. The GRANA tool is over 100 times faster compared with currently used manual approaches. As a proof of concept, we have successfully applied GRANA software to diverse grana structures across different land plant species grown under various conditions, demonstrating the wide range of potential applications for our software. GRANA tool supports large-scale analysis of grana nanomorphological features, facilitating advancements in photosynthesis-oriented studies.

## Teaser

An artificial intelligence–based tool enables fast, quantitative analysis of chloroplast membrane architecture, advancing understanding of the role played by structural organization in photosynthetic efficiency in plants.

## Introduction

Granum is a fundamental structural unit of the thylakoid membrane network specific for land plant chloroplasts (summed up in, e.g. Gunning and Schwartz 1999; Mullineaux 2005; Pribil et al. 2014; Kirchhoff 2019; Mazur et al. 2021). It is built of thylakoids that are flattened vesicles consisting of a protein–lipid–pigment membrane bilayer and an internal aqueous compartment known as a “lumen” (lately discussed in Perez-Boerema et al. 2024). Granum is composed of numerous thylakoids forming a multitiered system in an approximately cylindrical shape connected to a helical network of stroma thylakoids (Mustárdy and Garab 2003; Austin and Staehelin 2011; Bussi et al. 2019). Thylakoids adjacent to each other in the stack are separated by a thin layer called the “stromal gap” (Anderson et al. 2012). Such a structure, in which the thylakoid membranes separate the lumen from the stromal region, is crucial for creating a proton gradient, which is critical in producing high-energy molecules during the light phase of photosynthesis (summarized, e.g. in Johnson 2016).

Although grana and stroma thylakoids are connected, their molecular composition is not uniform, particularly in terms of proteins. Appressed membranes of grana thylakoids are rich in Photosystem II (PSII), while the stroma-exposed membranes of grana and stroma thylakoids are dominated by Photosystem I (PSI) and ATPase complexes. A recent study by Wietrzyński et al. (2024) indicated that protein complex concentration is consistent across all regions of grana appressed membranes. Additionally, they found no variability in protein complex distribution between nonappressed regions, regardless of whether these regions belonged to grana or stroma thylakoids. The lateral heterogeneity in protein distribution between appressed and nonappressed regions is believed to result from the steric hindrance caused by large stroma-exposed parts of PSI and ATP synthase protein complexes that are highly abundant in the stroma thylakoids. Additionally, specific packing of LHCII and LHCII-PSII supports vertical interactions between adjacent thylakoid layers. Thus, spatial distribution, the packing, and vertical interactions of photosynthetic complexes are closely linked to the architecture of internal chloroplast membranes and play a critical role in their self-assembly (Dekker and Boekema 2005; Anderson 2012; Garab 2016; Ruban and Johnson 2015). This allows for indirect inferences about the content and proportions of photosynthetic complexes based on the nanomorphology of thylakoid membranes.

Typically, the diameter of a granum ranges between 300 and 600 nanometers (Dekker and Boekema 2005; Bussi et al. 2019; Höhner et al. 2020). However, other lineages of vascular plants, such as ferns, cycads, and lycophytes, may exhibit wider grana with larger diameters (Bonatti and Fornasiero 1990; Nasrulhaq-Boyce and Duckett 1991; Colpo et al. 2023). Regarding the height of a granum, there is no upper limit to the number of membranes that can make up the stack. Nonetheless, there is ongoing discussion about the minimum number of thylakoids required to be considered a granum. Typically, a granum consists of 5 to 25 thylakoid membranes; however, there are also so-called “giant” grana stacks composed of even over 50 membranes (Anderson et al. 1973; Chow et al. 2005; Belgio et al. 2015). Researchers also observe 2-membrane stacks referred to as “membrane overlaps” (Gunning and Steer 1975), “doublet thylakoids” (Allen et al. 1988), “thylakoid doublets” (Ferroni et al. 2020), or “stacked thylakoid doublets” (Garty et al. 2024). Such configurations are frequently present, for instance, at the very early stages of chloroplast development (Kowalewska et al. 2016; Armarego-Marriott et al. 2019; Pipitone et al. 2021) but were also reported in mature chloroplasts of certain wheat (*Triticum* spp. L.) chlorophyll-deficient mutants (Allen et al. 1988; Aliprandi et al. 2024).

**Figure 1.**
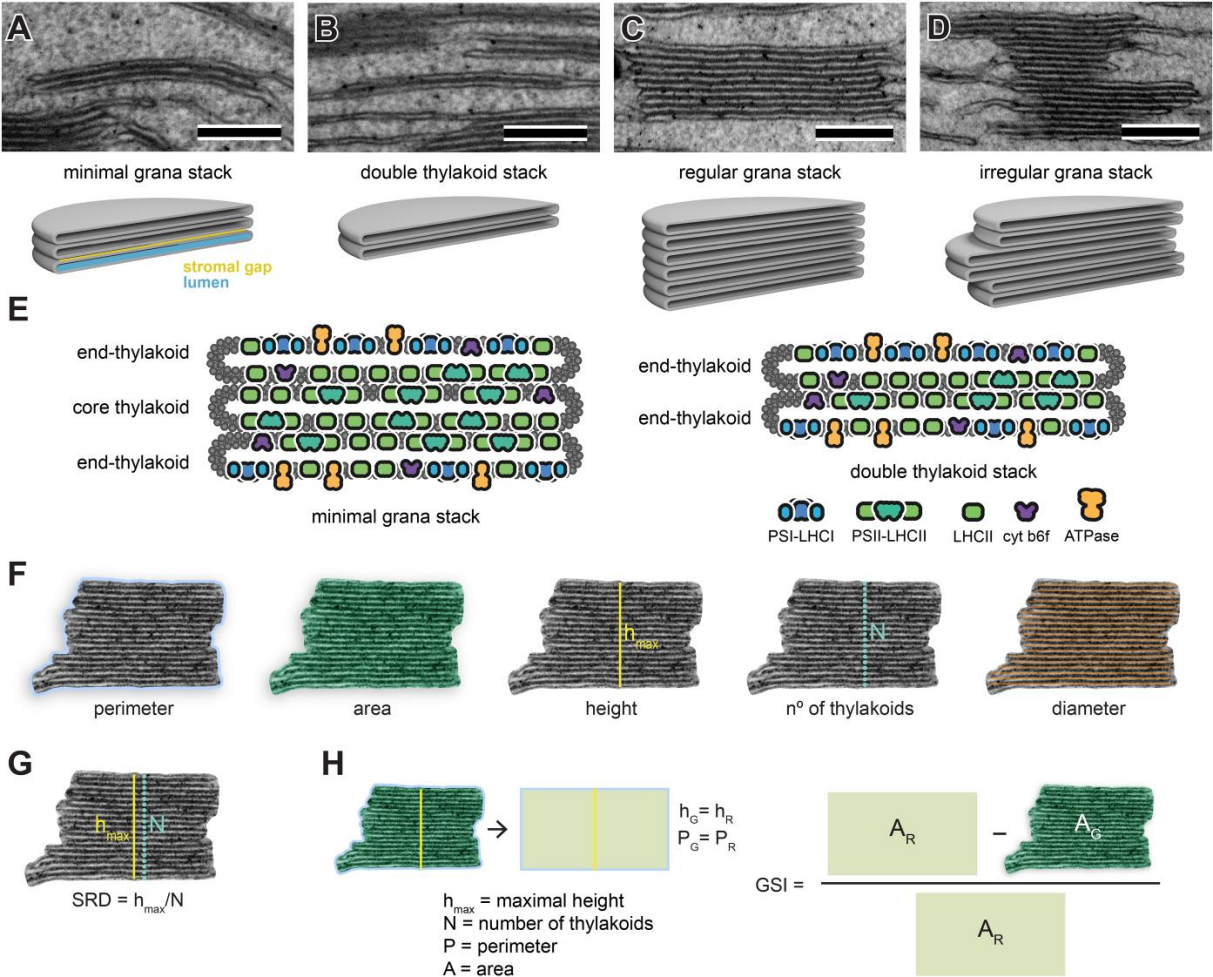
Ultrastructural characteristics of stacked thylakoid membranes. **A** to **D)** The structural variability of stacked thylakoid membranes; note that double thylakoid stacks cannot be considered grana due to the absence of 2 key membrane types: end and core thylakoids (shown in **E)**. **F)** A visual representation of the manual annotation of basic grana structural parameters obtained from TEM images. **G)** A visual description of SRD parameter manual calculation. **H)** GSI parameter definition; scale bar = 200 nm **(A** to **D)**.

In terms of molecular composition, a granum stack consists of its “core” (thylakoids appressed at both sides), edges referred to as “grana margins” [which include the curved regions at the termini of grana thylakoids also referred to as “curvature” (Trotta et al. 2019) and the transition regions between grana and stroma thylakoids], and the most external thylakoids in the stack called “end thylakoids” (reviewed in Ruban and Johnson 2015; Koochak et al. 2019). As mentioned above, core thylakoids of the grana stack are molecularly homogeneous. At the same time, end thylakoids have a heterogeneous character—the stromal gap-exposed part is similar to the core thylakoids, while the outer part (end membrane) to stroma lamellae (Anderson et al. 2012; Fig. 1E). Consequently, the lowest granum stack, containing both thylakoid types, must have a minimum of 3 layers—2 end thylakoids and 1 core thylakoid (Mazur et al. 2021; Fig. 1, A, B, and E). Interestingly, although early works reporting double membrane stacks (Gunning and Steer 1975) were published before molecular heterogeneity of grana thylakoids was reported, the authors already distinguished them from typical grana stacks based on structural premises alone.

Grana rarely forms perfect cylinders (Fig. 1, C and D). Shifting of thylakoids in the lateral plane can result in the formation of complicated structures for which multiple heights can be determined. As proposed earlier by us (Mazur et al. 2021), we suggest subdividing such arrangements into separate granum stacks, taking into account the boundary conditions that define the minimum number of membranes (2 terminal thylakoids and 1 core thylakoid) constituting a granum.

A precise comprehension of grana structure, combined with the molecular composition of its membranes, allows for establishing reliable boundary conditions describing these structures. This is essential for achieving results that are comparable across different research studies.

Due to the grana nanometric scale, the use of high-resolution electron microscopy techniques is essential for the detailed analysis of grana thylakoid morphology. In general, observations of chloroplasts are performed using fluorescence and electron microscopy (summed up in Kirchhoff 2019; Mazur et al. 2021). In the case of different fluorescence methods, observations are mainly based on detecting chlorophyll autofluorescence in vivo at room temperature (reviewed in Kowalewska et al. 2019). In such conditions, red fluorescence spots seen in confocal laser scanning microscopy correspond mainly to the appressed grana thylakoid membranes containing LHCII-PSII supercomplexes rather than to nonappressed thylakoids containing mainly PSI (Mehta et al. 1999; Hasegawa et al. 2010). By using this method, information describing the entire network can be obtained, such as the distribution and number of granum stacks per chloroplast (Herbstová et al. 2012; Chen 2014; Wood et al. 2018, 2019; Mazur et al. 2019) and as for individual grana—the average size and diameter of granum (Uwada et al. 2017; Wood et al. 2019; Bykowski et al. 2021; Hepworth et al. 2021). Recently, the fluorescence expansion microscopy method has been applied to visualize the thylakoid network of spinach. By the expansion of the isolated membranes and their staining with a fluorescent dye, it was possible to visualize the detailed structure of grana and their connection with stroma thylakoids. However, this method has so far been successfully applied for 2 species only and requires the isolation of de-enveloped chloroplasts (Bos et al. 2023; Berentsen et al. 2025). Nevertheless, even higher resolution is obtained using transmission electron microscopy (TEM) by prior fixation of the whole leaf tissue.

Leaf samples for TEM can be acquired via chemical fixation together with its variation called microwave fixation (Li et al. 2020) or high-pressure freezing combined with the freeze-substitution method (e.g. McDonald 2014; Armarego-Marriott et al. 2019). The majority of published data are still obtained through chemical fixation, as it does not require specialized equipment and has the highest success rate. Using the TEM method, it is possible to observe individual thylakoid membranes in the stack and obtain parameters such as granum area, perimeter, diameter, height, and number of thylakoids building a stack (Fig. 1F). It is also possible to acquire more complex parameters, enabling indirect inference about the photosynthetic efficiency of thylakoid membranes, such as the stacking repeat distance (SRD) parameter and Granum cross-sectional irregularity (GSI) parameter described in detail below (Figs. 1, G and H). Throughout this paper, we will refer to the SRD parameter interchangeably as the period of a granum for ease of reference. Although TEM analysis allows for obtaining numerous structural parameters of grana, it is important to remember that in classical form, this method facilitates the analysis of 3D objects in 2D. When analyzing spatially complex structures in 2D, it is important to keep in mind certain limitations inherent to such an approach. The fixed samples are randomly sectioned, and it is impossible to determine whether a specific cross-section illustrates the diameter or another chord of the granum. As a result, measurements of grana diameters from TEM 2D sections are associated with high Sd. Drawing reliable conclusions is possible only after analyzing large datasets. Consequently, projects analyzing the ultrastructure of thylakoid membranes often span over several years to get a sufficient number of measurements.

Another approach to chloroplast inner membrane network analysis is cryo-electron microscopy (Cryo-EM). However, Cryo-EM focuses on the molecular level, taking into account the arrangement of individual protein complexes within the membrane, typically without delving too deeply into the nanomorphological level of thylakoid network arrangement (Charuvi et al. 2016; Wightman 2022).

To obtain a 3D image, electron tomography or focused ion beam scanning electron microscopy can be performed both for room temperature (RT) and cryo methods. Still, it requires several additional postprocessing steps, including, e.g. alignment, reconstruction, segmentation, and modeling (reviewed in Daum and Kühlbrandt 2011; Asano et al. 2016; Otegui and Pennington 2019; Staehelin and Paolillo 2020). Consequently, such methods, although recently machine learning (ML)-enhanced (Moebel et al. 2021; Lamm et al. 2022; Yamauchi et al. 2024), are time-consuming and prevent acquiring a large number of repetitions. From this standpoint, classical RT 2D TEM allows for the effective integration of detailed nanomorphological analysis while also facilitating the acquisition of a substantial number of biological replicates, supporting robust and reliable results. However, it should be stressed that large datasets also require complex quantitative analysis for grana ultrastructure. The challenge in such an approach arises from manual measurements, which lead to variations in published data due to the utilization of different measurement protocols and variability in the experience of a particular researcher. Manual measurements are also time-consuming, which frequently results in the use of small sample sizes. Furthermore, the set of employed structural parameters is often limited, and different researchers use various selections of them, preventing reliable cross-analysis of data published by different authors (in detail discussed in Mazur et al. 2021).

In this work, we present GRANA (Graphical Recognition and Analysis of Nanostructural Assemblies), an artificial intelligence (AI)-based software tool for automated and reliable analysis of grana nanomorphology. Our tool can be used in 2 key ways: first, it offers a fully automated approach utilizing *shape estimation* and *measurement modes*, where we employ 3 artificial neural network (ANN) models for image data processing along with mathematical formulas for calculating additional grana structural parameters. Second, GRANA supports the hybrid intelligence (HI) approach by providing output data in a nonblack-box format, carefully designed for efficient control and filtering of results, reducing the risk of errors typically associated with fully automated methods.

Our priority was to ensure that the created tool is user-friendly and easy to navigate. This approach will reduce the entry barrier for data analysis and serve as another step toward more reliable and quantitative data analysis of grana structures. Modern electron microscope software, along with motorized stages, enables efficient data collection across entire grids at specified magnifications (Zheng et al. 2010; Tan et al. 2016). The bottleneck in analyzing large volumes of images still is the time-consuming manual measurements. Our solution supports the automation of data analysis. Automatic grana detection according to specified boundary conditions allows for large, less biased, high-throughput analysis and interpretation of structural data also in light of biochemical and molecular studies.

## Results

This section presents essential information for GRANA tool users, including an overview of the tool's graphical user interface (GUI). The Materials and Methods section and the corresponding Supplementary materials describe technical details regarding the architecture and training of ANNs and the validation of the implemented solutions.

### Machine learning and software solutions

Our tool utilizes 3 ANN models for image data processing. To maintain brevity and clarity, we will use the terms ANN, model, and network interchangeably when referring to specific ANN models, with the latter 2 terms specifically denoting models with fixed parameters after training.

**Figure 2.**
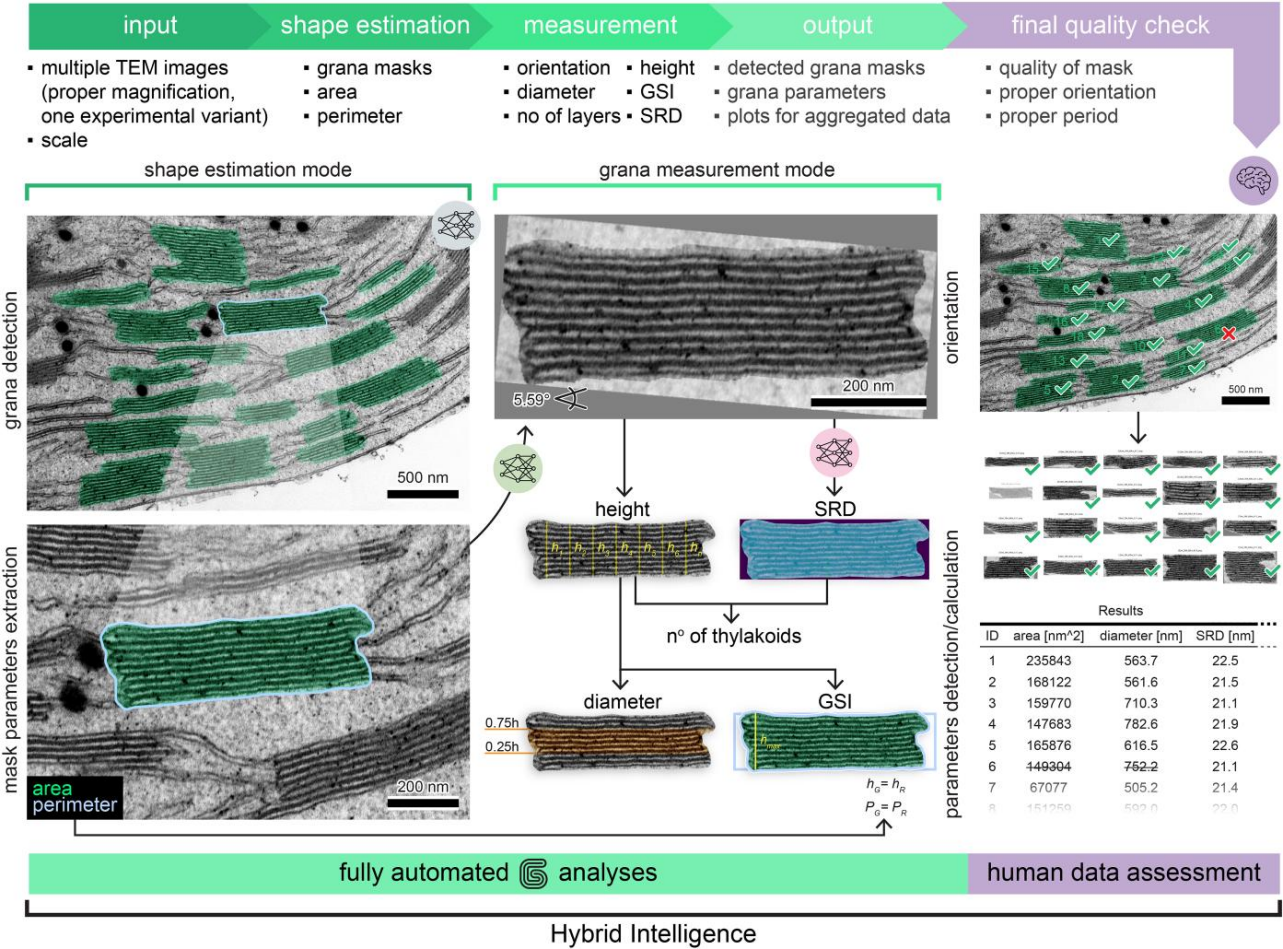
GRANA tool workflow—fully automatic and HI approaches. The GRANA tool consists of 2 modules (*shape estimation* and *measurement*), utilizing 3 ANNs (shape ANN—gray, orientation ANN—green, and period ANN—pink) to automatically annotate grana on TEM images and calculate structural parameters (area, perimeter, height, diameter, thylakoid number per granum stack, SRD, and GSI). GRANA can simultaneously analyze multiple TEM images of thylakoid networks captured at the same magnification. The tool's output provides graphical and numerical data, enabling rapid quality checks of automatically generated results and facilitating HI-based analysis.

Deterministic algorithms are implemented to calculate the remaining parameters based on the data previously processed using ANNs. The GRANA workflow is divided into 2 modes: *shape estimation mode*, in which grana thylakoids are recognized and labeled, and *measurement mode*, in which the structural parameters describing the nanomorphology of grana are determined (Fig. 2). The *shape estimation mode* uses 1 ANN model, while the *measurement mode* utilizes 2 remaining models. All 3 ANNs are integrated into the GRANA software pipeline in such a way that the user is unaware they function independently, although their training was conducted separately.

The ANN we use to estimate grana shape is a YOLO (which is an acronym for You Only Look Once) model (Redmon et al. 2016) trained to detect and label grana thylakoids on TEM images. In the following, we will refer to this network as the shape ANN. From this step, mask parameters, such as granum area and perimeter, are extracted. Granum area represents the surface area of the polygon corresponding to the cross-section of the granum stack in the TEM image, while the granum perimeter is the outline of this polygon, referring to the length of the nonstacked membranes of the grana (end membranes and edges of grana margins) (Figs. 1F and 2). Both are essential for calculating the complex parameters described below. Only fully visible grana in the image are masked and taken into account when generating structural parameters. Moreover, it should be noted that detailed analysis of all granum structural parameters in GRANA is possible only for samples in which grana stacks are visualized parallel to the vertical granum direction—this, in principle, is obtained by cross-sectioning the leaf blade.

After estimating the granum mask, its numerous sections (patches) are extracted and augmented through random rotation and scaling. The orientation estimator is a ResNet18 network (He et al. 2016) fine-tuned to recognize the entire grana orientation from extracted sections (a detailed description of the working principle and training of the orientation ANN is provided in the “Orientation ANN” section in Materials and methods— and Supplementary Data S1). This specialized ANN will be referred to as the orientation ANN. Finally, refinement of the orientation estimation is performed by identifying the angle that minimizes the area of the bounding rectangle enclosing the granum mask (Fig. 2). After estimating the granum orientation, it is possible to determine the remaining grana structural parameters, including height, diameter, GSI, SRD, and the number of thylakoids. Granum height is the distance between the upper and lower layers of the end thylakoids, and it is manually established as the longest segment along the vertical axis of the stack (Fig. 1F). Our tool determines this by measuring the multiple heights of the granum in each column of the mask (the distance between the highest and lowest pixel). Then, the 0.8 quantile of the highest height values is returned as the final result (Fig. 2). After establishing the height, it is possible to define the mask region along the horizontal axis, where the diameter of the granum stack will be measured. Within the middle 50% of granum height, the horizontal extent of the granum is calculated as the distance between the rightmost and leftmost pixels of the mask. Subsequently, an average value of the horizontal extent (granum diameter) is provided (Fig. 2). Manually, the diameter is determined by measuring the length of each layer building the granum and calculating their average value (Fig. 1F).

Knowing the granum area, perimeter, and height, it is possible to calculate the GSI parameter. It describes the irregularity of the granum cross-section by comparing the granum area to the area of a rectangle with an identical height and perimeter as a particular granum (Fig. 1H). The higher the GSI parameter, the more substantial the shift of the thylakoids in the stack relative to each other in the horizontal plane. Both manually and in the GRANA tool, GSI is established using the exact definition (see formula in Fig. 1H). It is worth noting that a similar parameter, granum lateral irregularity (GLI), was introduced earlier by our group (Kowalewska et al. 2016). GLI as a relative variation provides a measure of irregularity based on thylakoid diameter variation. However, it does not account for thylakoid shifting in the lateral plane. Therefore, we introduced GSI, now embedded in the GRANA tool, to provide more comprehensive structural information about GSI, capturing both diameter variations and lateral displacement of thylakoids within the stack.

Another parameter calculated within the GRANA is SRD, which represents the average thickness of thylakoids in the stack together with the neighboring stromal gap. Manually, it is measured by the height value divided by the number of thylakoids (Fig. 1G). In our method, we employ a custom-built period estimator: a convolutional neural network (CNN) (Lecun et al. 1998) with an asymmetric receptive field enhanced with an attention mechanism (Bahdanau et al. 2016) that targets overlapping sections of the input image. These sections are encoded with a regularized representation, leading to the final prediction. This ANN will be referred to as the period ANN (a detailed description of the working principle and training of the period ANN is provided in the “Period ANN” section in Materials and methods and Supplementary Data S2). The final parameter generated by the GRANA tool is the number of thylakoids building each grana stack (Fig. 1F). In our tool, it is calculated by dividing the granum height by its SRD parameter value (Fig. 2).

### Tool overview

The GRANA software is available as a Docker container that can be run on a personal computer and as a demo version on the Hugging Face platform. The demo version has full functionality, limiting only the number of images that can be analyzed simultaneously to 5. Both the full and demo versions are available on the chloroplast.pl/GRANA website. To test the functionality of both versions of GRANA, one can use a sample dataset available at the aforementioned address. The guide available at github.com/center4ml/GRANA will be helpful in installing our tool on a PC.

The GRANA interface is divided into 2 main sections: input, which includes all the information provided by the user, and output, which consists of the results returned by the tool (Supplementary Fig. S1). Each interface section is accompanied by a brief textual description to enable easy and efficient utilization of the tool's capabilities.

The input section includes a data upload window at the top and a scale setting area below. In the data upload window, users can drag and drop .tiff, .png, and .jpg files or click to select and upload images from a folder. It is essential to remember that the quality of the input data determines the quality of the output results (Supplementary Fig. S2). Uploaded TEM images should be of the same scale and represent the same experimental variant to obtain reliable results. Micrographs with around 5 to 30 grana stacks visible in 1 frame should be selected to acquire the full range of structural parameters. The scale setting module consists of 3 text boxes. One can either provide the pixel per nanometer ratio (on the left) or the length of the scale bar in pixels and nanometers (on the right) to have the ratio calculated. After uploading files and setting the scale, users must initiate the analysis by clicking the “Submit” button, which locks for the duration of the analysis and displays a message indicating that the data are being processed.

After the necessary processing time has elapsed (dependent on the number of uploaded files), the output section components are displayed on the screen. The top section lists all the available results and contains the “Download results” button. The next output section is a navigable gallery of all uploaded TEM images with masks of recognized grana structures. Each granum mask is labeled with its number corresponding to the numbers visible in the following contact sheet of individual grana and the “granum ID” column in the table with all raw measurements.

Below, a table of aggregated results for all uploaded TEM images is displayed; as such, our software enables quick, preliminary analysis of results without requiring the user to process raw data. This is further facilitated by violin graphs illustrating aggregated values for selected parameters.

The following output section is the contact sheet of individual rotated grana detected on uploaded images with their unique names composed of the granum number and file name. Both displayed galleries are crucial for the reliable assessment of automated GRANA output in the HI approach.

Lastly, a table containing all raw data for each granum is provided. After downloading, a user can filter the obtained data as desired.

Both displayed tables, annotated images, and single grana contact sheets can be downloaded in .zip format by pressing the “Download results” button. Output data are organized to facilitate the identification of specific grana stacks on masked images, rotated grana contact sheets, and in the results table. This organization enables rapid quality checks of obtained results, which can be performed by a human expert (HI approach). While in many cases, such data filtering is unnecessary due to GRANA's high accuracy (described below), this capability becomes crucial when input data quality issues arise, ensuring the acquisition of reliable results.

### Manual, automated, and HI-based approaches: the advantage of human and machine cooperation

To evaluate the functionality of the current version of the GRANA tool and its capability of providing reliable values of grana structural parameters, we analyzed cultivated tobacco (*Nicotiana tabacum* L.) TEM images (Supplementary Tables S1 and S2) using the GRANA tool's fully automated and HI-based approaches, and we contrasted it with purely manual measurements performed in ImageJ software (Schneider et al. 2012). Cultivated tobacco was selected for this purpose because none of the ANN models implemented in the GRANA tool had been trained on grana from this species.

We measured the time required for the analysis (i) performed by the GRANA software (run on a system equipped with a 3.5 GHz 64-bit processor, 4 cores, and constrained to a 4 GB RAM limit), (ii) the additional time needed for HI-based approach, in which the expert filtered automatic results using GRANA output data, and (iii) done by an expert highly experienced in manual measurements.

We showed that the utilization of the GRANA tool automatic mode accelerates the analysis process by a factor of a hundred (from 443 to 4 min for the tested example) and only an additional 25 min of expert work was required for data filtering in HI solution for the tested dataset.

**Figure 3.**
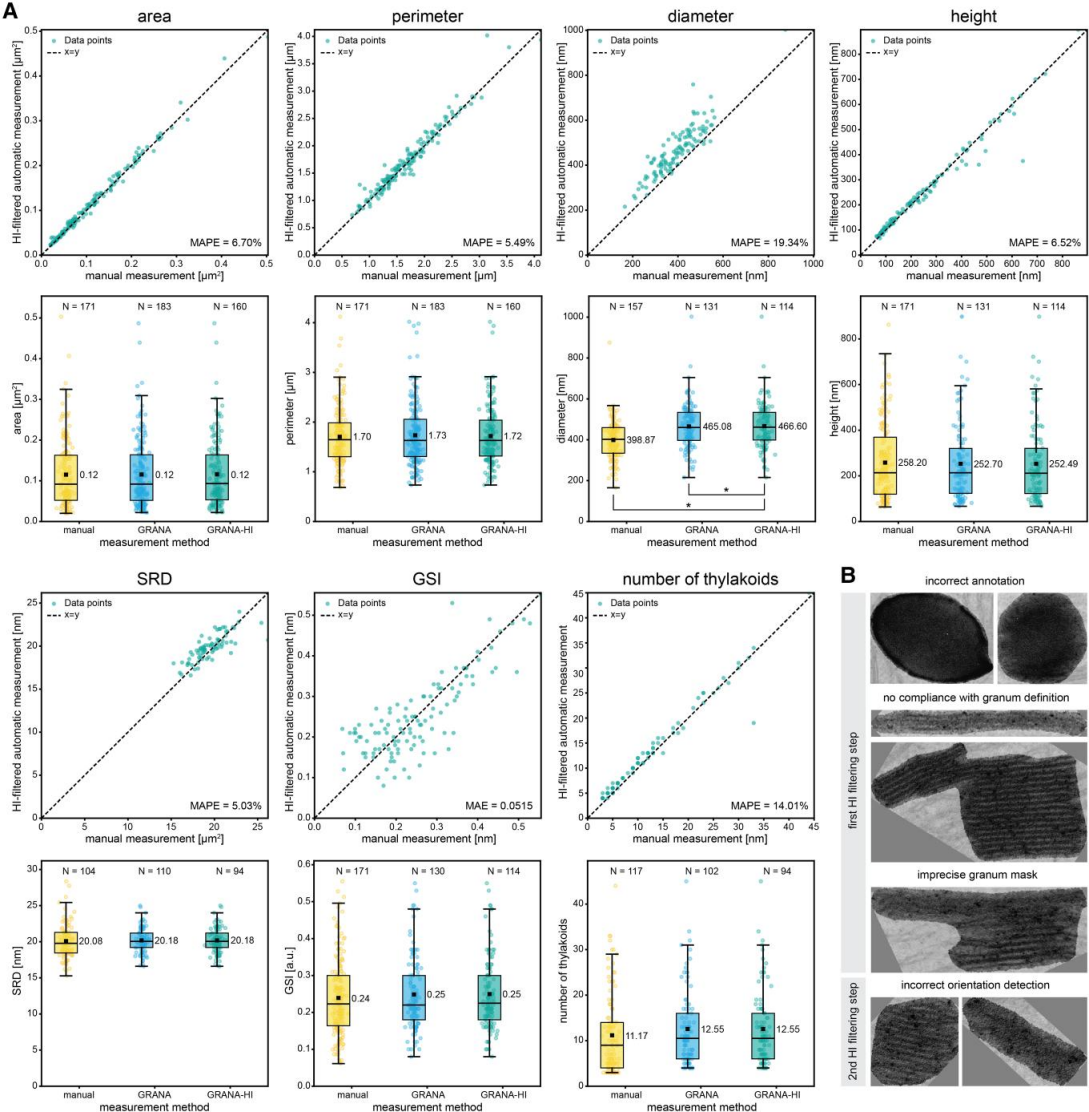
Comparison of manual, automated (GRANA), and HI-based (GRANA-assisted) approaches for measuring grana structural parameters. **A)** Scatter plots illustrating the correlation between manual and HI-filtered measurements for area, perimeter, diameter, height, SRD, GSI, and number of thylakoids parameters. The *x*-axis represents manual measurements, while the *y*-axis represents HI-filtered automatic measurements. Each green point visible on the scatterplots corresponds to an individual measurement. The MAPE values are shown on the bottom right of the plots for area, perimeter, diameter, height, SRD, and number of thylakoid parameters, and the mean absolute error (MAE) value is provided for GSI parameter (GSI is a ratio ranging between 0 and 1); under each scatter plot, a box plot comparing manual, automated, and HI-filtered measurements for each parameter is presented; the bottom and top of each box represent the first and third quartiles, respectively. The whiskers denote Sd, every point visible on the boxes represents individual measurement. A horizontal line on the box plot indicates the median value, and a square represents the mean value which is also displayed as a numeric result to the right of the box. No statistically significant differences were detected between the measurement methods at *P* = 0.05 (1-way ANOVA with post hoc Tukey test) for all parameters except diameter, which likely results from the use of different calculation methods in GRANA compared with those employed by human operators (Figs. 1 and 2). **B)** Examples of grana masks generated by the GRANA tool, which were rejected during the 2 steps of HI-assisted data filtering.

In the GRANA HI approach, final data filtering can be processed in 3 steps, in which operation of 3 ANNs is evaluated by the expert using visual GRANA output. In the first step, the human operator checks the quality of grana masks using the gallery of annotated TEM micrographs. In the second step, the accuracy of grana mask rotation can be validated using the contact sheet gallery of individual grana stacks (see Fig. 3B for examples). Finally, to verify the operation of the period ANN, the human operator can visually score the correctness of the recognized number of thylakoids building each stack. In our example, in total, the human operator filtered 12.6% of grana masks due to incorrect detection and <1% due to incorrect orientation detections. No major errors from the period ANN were observed in our dataset. To evaluate the precision of HI obtained results, we calculated mean absolute percentage errors (MAPEs) for analyzed parameters. The MAPE ranged from 5.03% for SRD to 19.34% for diameter (Fig. 3A). The relatively high MAPE and positive bias for diameter (Fig. 3A) are likely due to differences in the methods used for its calculation in GRANA compared with those used by human operators (Figs. 1 and 2). Specifically, the automated method implemented in GRANA determines the diameter only after establishing granum orientation by orientation ANN and subsequently defines the measurement region as the middle 50% of the granum's height. This approach ensures accurate diameter estimation even for grana that are not perfectly flat in the lateral direction. Within this region, diameter is calculated as the horizontal extent between the leftmost and rightmost pixels of the granum mask. However, even minor inaccuracies in orientation estimation can lead to slight rotations of the granum mask, causing the calculated horizontal extent to represent a chord rather than the true granum cross-sectional diameter, resulting in systematic overestimation. To mitigate this issue, we introduced a refinement step in the orientation workflow, rotating the granum mask within −10 and +10 degrees to identify the minimal bounding rectangle enclosing the mask. Despite this improvement, small residual errors persist due to the intrinsic difficulty of perfectly aligning mask orientation. It is important to note that, although the most precise manual approach involves averaging multiple diameter measurements across individual thylakoids (described in the “Machine learning and software solutions” section in Results and shown in Fig. 1F), this method is rarely employed by researchers due to its laborious nature. Typically, other studies rely on a single measurement across the central region of the granum stack (full width at half-maximum—FWHM). Consequently, our automated approach, averaging the result over 50% of granum height, is notably more accurate than a single manual measurement. The minor but systematic overestimation compared with measurements of all thylakoid layers does not affect the comparability of data obtained across different experimental conditions.

These results were further considered in a context of potential variation among different human operators. We calculated the MAPE for manual measurements performed by 3 human operators on the same dataset, using the height parameter as an example. The MAPE was calculated pairwise, and the average of the 6 pairwise comparisons was taken. We obtained an average MAPE of 8.85% between the measurements of the 3 human operators with individual MAPE values ranging from 4.54% to 12.18%. It is very similar to the MAPE between the HI-filtered results and human operators (6.52%, 6.43%, and 14.79%, with an average of 9.25%). These results indicate that the variation between HI-filtered and manual results for the height parameter is comparable with the variation among the human operators, suggesting that the obtained MAPE value presented in Fig. 3A can be considered an intrinsic result variability.

We also investigated whether fully automatic mode was significantly less precise than the HI approach. To evaluate this, we used the SRD parameter, as it is the only grana parameter that can be meaningfully averaged per TEM image. The calculated MAPE value on the per-image averaged SRD parameter for the comparison between fully automatic and manual modes was 3.55%, while for HI-filtered vs. manual, it was 3.45%. Such a small difference suggests that the results obtained through the fully automatic mode are not significantly inferior to those obtained using the HI approach for this dataset. The lack of statistically significant difference between manual, automatic, and HI-obtained data for all parameters, except for the aforementioned diameter, supports this hypothesis (Fig. 3A). However, it is important to note that HI filtering may be a crucial step when analyzing lower quality data.

Finally, we evaluated the data loss of GRANA-assisted HI measurement compared with manual measurements using the cultivated tobacco dataset (Fig. 3A). After the grana shape estimation step, 10% of grana detectable by manual methods were lost, reducing the number of grana masks available for calculating area and perimeter parameters. After the orientation estimation stage, around 30% of manually obtainable diameter (27.39%), height (33.33%) and GSI (33.33%) parameters did not have their corresponding results provided by the software. In the SRD value estimation, 17.5% of grana were unreadable, in comparison with 5% for the human operator. While these values represent a considerable data loss, it is important to note that for large datasets (*n* > 100), this loss is unlikely to affect the overall relevance of the results.

### Tool in action

**Figure 4.**
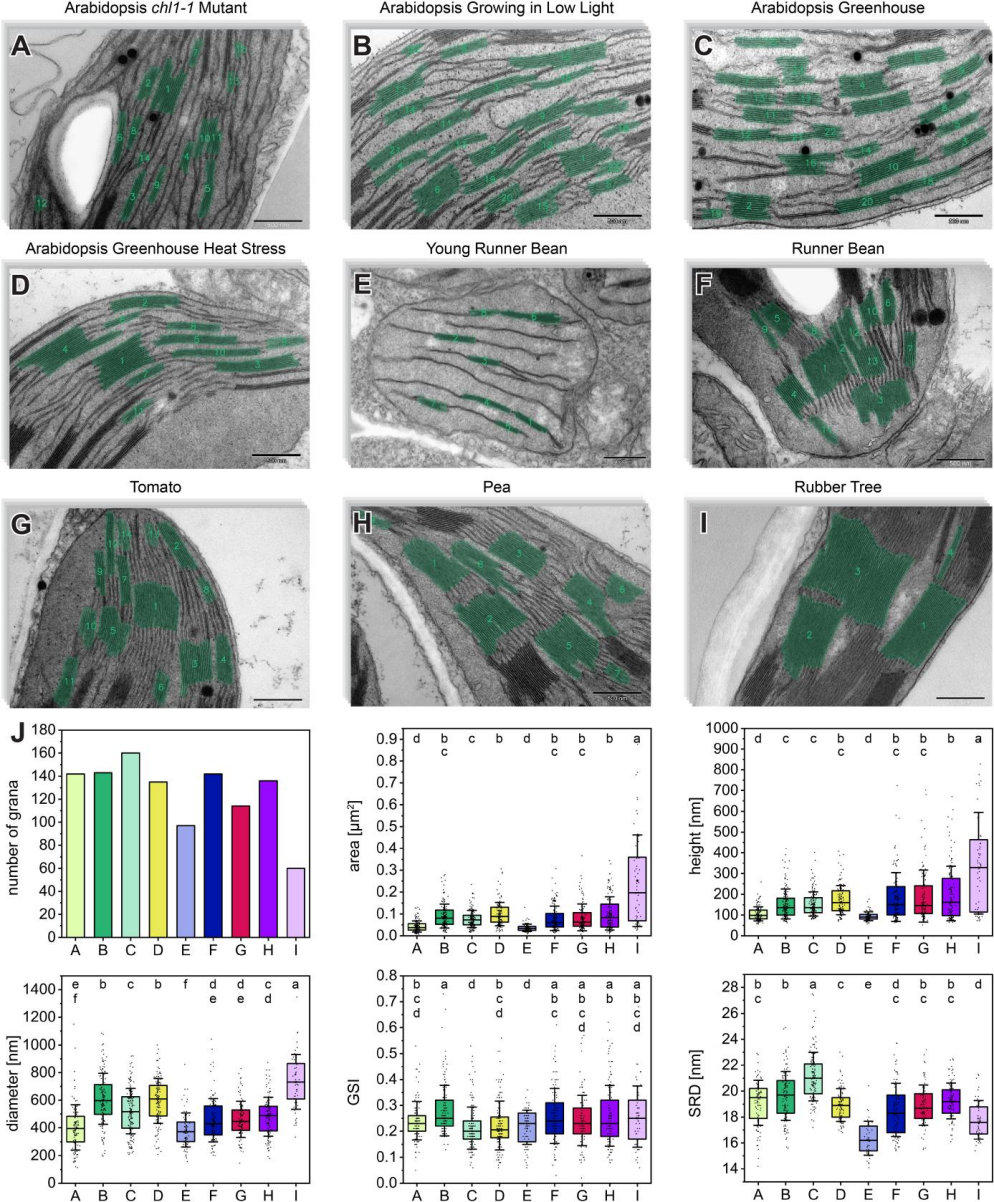
Quantitative analysis of ultrastructural parameters in various land plant species and experimental conditions obtained using GRANA. **A** to **I)** Exemplary images of 10-image stacks were analyzed for each variant. **A)** Arabidopsis mutant exhibiting small grana stacks (*chl1-1*). **B)** Arabidopsis grown under low light in a growth chamber. **C)** Arabidopsis grown in a greenhouse in control conditions. **D)** Arabidopsis grown in a greenhouse in control conditions and heat stressed for 24 h. **E)** Runner bean seedling grown for 2 d in a growth chamber in medium light conditions in optimal temperature. **F)** Mature runner bean grown as **E**. **G)** Mature tomato grown as **E**. **H)** Mature pea grown as **E**. **I)** Shade-tolerant rubber tree with characteristic giant grana stacks grown in greenhouse. **J)** Plots of parameters obtained in GRANA software for all analyzed pictures; the bottom and top of each box represent the first and third quartiles, respectively. The whiskers denote Sd, and every point visible on the boxes represents individual measurement. All quantitative data were generated in GRANA in <30 min of processing time; presented results were not filtered by the human expert. Pairs of results marked with different letters differ significantly at *P* = 0.05 (1-way ANOVA with post hoc Games–Howell test); scale bar = 500 nm **(A** to **I)**.

As a proof of concept, to demonstrate the functionality of the GRANA software, we present a set of structural parameters generated for various structural types of grana (Fig. 4). To demonstrate the tool's capabilities for recognizing and analyzing a wide variety of grana examples, 9 different datasets of TEM images were prepared, featuring grana from various land plant species or developing under different experimental conditions (see Supplementary Tables S1 and S2). Each set comprised 10 micrographs at the same magnification with 60 to 160 annotated grana stacks per variant. In analyzed samples, the average granum area ranged from around 0.04 to 0.2, with the smallest grana found in 3-d-old runner bean (*Phaseolus coccineus* L.) chloroplasts and Arabidopsis (*Arabidopsis thaliana* (L.) Heynh.) mutants characterized by decreased chlorophyll content and the largest in shade-tolerant rubber trees (*Ficus elastica* Roxb. ex Hornem, 1819). The diameter analysis indicated significantly wider stacks in Arabidopsis than in other species typically used in photosynthesis-related studies, such as runner bean, tomato (*Solanum lycopersicum* L.) and pea (*Pisum sativum* L.).

Interestingly, low light conditions resulted in increased grana irregularity in Arabidopsis compared with other growing conditions applied in this species. The value of the SRD parameter varied among the analyzed experimental sets, with the highest average value observed in Arabidopsis grown in RT and the lowest in young runner bean chloroplasts. The extensive range of values obtained for individual parameters demonstrates the considerable diversity of grana thylakoids, which can be species and growing condition specific. This necessitates large-scale grana nanomorphology studies facilitated using the GRANA tool to understand the observed patterns, e.g. recognizing mechanisms of photosynthesis regulation.

## Discussion

This work is a reference paper for the open-source GRANA tool, an AI-enhanced software tailored for fast, comprehensive, and quantitative ultrastructural analysis of grana thylakoids in land plant chloroplasts. The current version of the GRANA is available on github.com/center4ml/GRANA. New versions of the tool will be available at the same address.

In this work, we have demonstrated the functionality of the GRANA tool using a set of micrographs of various land plant thylakoids. We have also compared the automatic methods based on ANNs, an HI approach, and standard purely manual measurements performed by a human expert. Using the tobacco thylakoid dataset as an example, we have demonstrated that the application of our tool reduced the time required for analysis by a factor of a hundred, and the HI approach secures high-quality results. It should be noted that the method was primarily developed and validated using angiosperm samples. While GRANA can be applied to other plant groups, users should exercise special attention when analyzing sister lineages to seed plants (*Spermatophyta*) or specimens with atypical grana morphology, as some structures like long thylakoid triplets or oddly shaped grana may require additional verification.

Utilizing the GRANA tool will lower the entry threshold for starting chloroplast ultrastructural analyses by researchers, who have frequently treated TEM images more as illustrations of research material rather than valuable and quantitative structural data. Consequently, the GRANA tool facilitates the seamless integration of ultrastructural analyses into a broader dataset acquired through diverse methods by a specific research team. GRANA also reduces the need for an expert to analyze the structural data of thylakoid networks, as the intuitive functionality allows one to navigate through key measurement stages. The next step is simply filtering the data according to expectations if one decides to use an HI approach or stick to a fully automated workflow. The unified measurement protocol embedded in the GRANA tool enables the generation of results in a standardized workflow, allowing for comparisons between different research groups, which has not been possible until now. Employing the GRANA tool is a step toward generating a large amount of data that can be used in meta-analyses and, e.g. for creating innovative ultrastructural databases in the future. Unified research methods will enhance the importance of such data and enable their application in multicomponent analyses regarding composition, evolutionary background, and developmental stages.

Currently, the ultrastructural level of thylakoid organization itself raises interest due to the interaction of grana structures with light, which hints at the important role of grana nanomorphological features in the modulation of photosynthetic efficiency (Johnson and Wientjes 2020). In specific conditions, nanophotonic effects, which give rise to structural color, may also occur. Such a phenomenon can be observed in shade-adapted species of begonia (*Begonia* spp. L.) or spikemosses (*Selaginella* spp. P. Beauv.), where thylakoids are organized in planar multilayered structures within iridoplasts and bisonoplasts, respectively, exhibiting photonic crystal properties (Jacobs et al. 2016; Masters et al. 2018). Moreover, Capretti et al. (2019) demonstrated that grana are effective nano scatterers of visible light and function as biological equivalents to dielectric nanoparticles. Such interdisciplinary research highlights the importance of thylakoid nanomorphology as one of the crucial yet frequently neglected levels of photosynthetic efficiency tuning.

The introduction of our tool is also timely since the current development of open science is associated with the necessity of depositing raw data in open-access repositories, as required by national regulations and journal policies. In this context, reusing and reevaluating data will be accessible, even if collected by different research groups. Therefore, there is an increasing demand for standardized analysis tools that enable data comparison unaffected by human bias.

The GRANA tool is a ready-to-use software that is user-friendly for inexperienced researchers. Yet, it enables obtaining valuable raw data that can be utilized by experts in the HI approach. Despite its simple interface and intuitive functionality, nothing is hidden from the user. The data obtained using the GRANA tool will enable the acquisition of valuable information about the ultrastructure of grana thylakoids, which can be used both in basic research as well as applied science aiming to develop, e.g. bioinspired optical materials or artificial photosynthetic systems (Li and Han 2018; Capretti et al. 2019).

## Materials and methods

### TEM sample preparation and visualization

TEM images of thylakoid network ultrastructure were obtained for various land plant species cultivated in different experimental conditions (details listed in Supplementary Table S1). Chemical fixation of ∼8 mm^2^ leaf samples for TEM was carried out in 2.5% glutaraldehyde in a 50 mmol L^−1^ cacodylate buffer (pH 7.4). Samples were postfixed with 2% OsO_4_ at 4 °C, followed by dehydration in a graded series of acetone and subsequent infiltration in acetone mixtures (3:1; 1:1; 1:3). Finally, leaf samples were embedded in a pure resin medium (Agar 100 Resin Kit). Obtained blocks with samples were cut into ultrathin specimens with a thickness of ∼70 to 90 nm using the ultramicrotome (Leica UTC). TEM studies were performed in the Laboratory of Electron Microscopy of the Nencki Institute, supported by the project financed by the Minister of Education and Science based on contract no. 2022/WK/05 (Polish Euro-BioImaging Node “Advanced Light Microscopy Node Poland”).

### Dataset collection and labeling for tool development

The datasets used for ANN training consisted of TEM micrographs illustrating the thylakoid network of chloroplasts from various land plant species grown under different developmental conditions (Supplementary Tables S1 and S2). Some data were collected specifically for this project, while the majority were gathered in our research group over the past several years. TEM images selected for the training represented chloroplasts of Arabidopsis, runner bean, pea, rubber tree, rose mallow (*Hibiscus × rosa-sinensis* L.), spider plant (*Chlorophytum comosum* (Thunb.) Jacques), and variants of these plants grown under chilling and heat stress conditions to create highly diverse training sets. Therefore, these sets consist of young and mature plants, wild-type and thylakoid composition mutants, model and crop species, monocots and dicots, as well as plants acclimated to or stressed in different conditions known to influence grana structure. We used micrographs collected using different CCD cameras and photographic films to increase the technical variability of the analyzed images.

### GRANA shape estimation mode

#### Shape ANN

The shape ANN is based on the YOLO algorithm, which takes an image as input and uses a deep CNN to detect objects within the image. YOLO is a 1-stage model that simultaneously predicts both the presence and location of objects. YOLOv8 offers various models with different trade-offs between accuracy, speed, and model size (Jocher et al. 2023). For our purposes, we selected the YOLOv8s variant, a smaller model that balances speed and accuracy effectively. The YOLOv8 architecture comprises 3 main sections: the backbone, neck, and head. The backbone is the deep learning architecture that extracts features from the input image. The neck combines features from different backbone layers. Finally, the head predicts the bounding boxes and masks of the objects, which are the final outputs of the shape estimation model.

#### Shape ANN training

**Figure 5.**
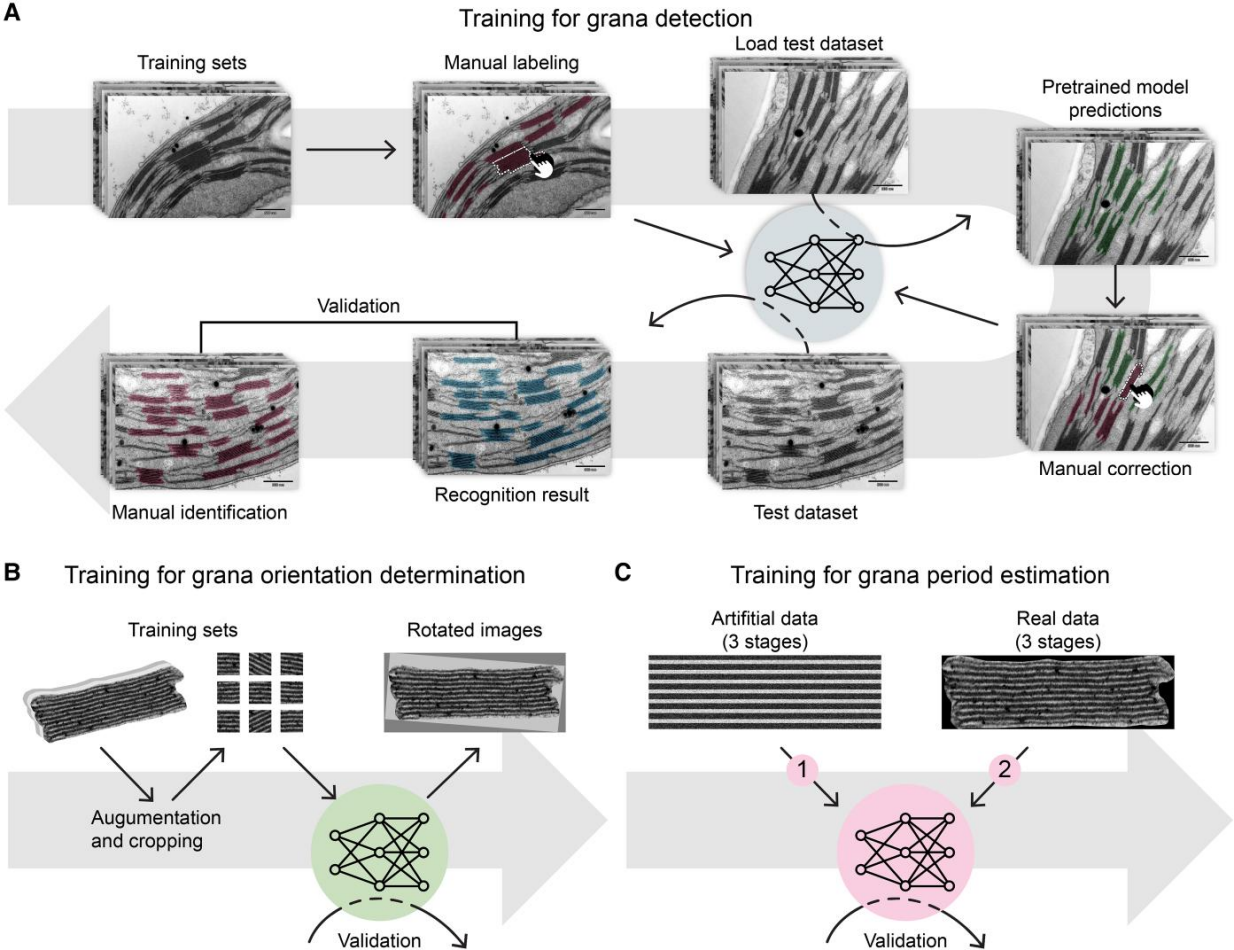
Graphical summary of the training procedures applied for 3 ANNs employed in GRANA tool. **A)** Representation of the shape ANN training, which included (i) manual labeling of grana thylakoids on TEM micrographs (training dataset), (ii) implementation of the training dataset into the YOLO model, (iii) generation of predictions on a test dataset by the pretrained YOLO model, (iv) manual correction of model predictions (ii to iv repeated 4 times), and (v) validation of the obtained recognition results through comparison with manual annotations. **B)** Representation of the orientation ANN training which involved (i) the extraction and augmentation (through random rotations and scaling) of multiple square sections (patches) from grana masks with manually determined orientations, (ii) feeding the patches into the Resnet18 model, and (iii) validation of the rotated masks (90% of the dataset was used for training and 10% for the validation). **C)** Representation of the period ANN training which consisted of 3 stages using artificial data (1), including attention training, 3 stages using real data (2), with 70% of the dataset used for the training using real data and 30% for the validation and final evaluation of obtained results on dataset consisting of grana masks with manually determined SRD parameter by 4 human experts.

To train the shape ANN (Fig. 5A), grana thylakoids were manually annotated on micrographs by overlaying polygon-shaped masks. Through this process, 5 training sets (A to E) were created, consisting of 283 TEM images with 5,640 annotated grana (Supplementary Table S2). The ground truth annotations for grana in training Datasets B to E were manually created by refining the predictions generated by the YOLO model, which had been trained on previously annotated sets (i.e. for annotating Dataset B, the predictions from the YOLO model trained on Dataset A were used as the starting point; for annotating Dataset C, the predictions from the YOLO model trained on Datasets A and B were used as the starting point, etc.). The models were trained with image resizing and default data augmentation implemented in YOLO. During each stage of training, we consistently increased the number of images used.

#### Shape ANN selection and evaluation

Before we finally selected the YOLOv8s model for the shape estimation module, we tested and automatically validated 6 different models (A to F) with various architectures (Supplementary Table S3). We considered 3 metrics based on manually annotated images from the test dataset (20 TEM images): precision, recall, and F1 score (for details, see Supplementary Data S3).

In order to compare and evaluate the shape ANN predictions, the intersection over union (IoU) metric was calculated (see Supplementary Fig. S3 and Data S3) for grana masks, represented by polygons. The manually labeled polygons from the test set served as the ground truth, while the model generated predicted polygons. A higher IoU value indicates a closer match between the ground truth and the predicted granum shape. Precision, recall, and F1 score were calculated over a range of IoU thresholds for 6 evaluated models (see Supplementary Tables S3 and S4). A minimum 40% overlap between the ground truth and predicted polygons was required for a prediction to be considered a *true positive*. Based on these results, Model D (YOLOv8s) was selected for grana detection.

### GRANA measurement mode

#### Manual labeling for measurement module training

In the selected TEM images, the ground truth values for the height, rotation angle of the grana, and the length (diameter) of the membranes forming the stacks were manually determined in ImageJ software. The rotation angle was directly derived from the measurement of the respective grana's height. This entire range of parameters was used as labels in training data for the individual functional modules of the tool—orientation and period ANNs (Fig. 5, B and C).

#### Orientation ANN

Grana orientation is estimated using a CNN ResNet18 that processes a batch of square sections (patches) of the granum. Each patch is subjected to test-time augmentation involving random rotation and resizing. The grana orientation estimate is calculated as the mean rotation of the patches, with the Sd of patch-level estimates used for certainty estimation.

The network's output is structured as a 2-number output, which represents a point in a 2D space corresponding to the estimated orientation. This choice naturally lends itself to the use of a specialized angle-prediction head. To address the inherent 180° symmetry of object orientations, we incorporate a 2-argument arctangent function, which computes the angle in the correct quadrant based on the 2 input values. This method ensures that the orientation estimate respects the cyclic nature of rotational symmetries. The result of the arctangent function is divided by 2, effectively constraining the output to a consistent orientation between 0° and 180°.

For the final orientation determination, beyond the ResNet18 network trained on our data, we employed subsequent refinement by minimizing the area of the bounding rectangle enclosing the granum mask, within −10° and +10° of the orientation ANN estimation.

#### Orientation ANN training

To train the orientation ANN, we used a dataset of 137 grana masks with manually determined orientation (Supplementary Table S2). Approximately 122,000 square sections of grana masks (up to 1,024 per granum) were extracted and augmented through random rotations and scaling. These sections were used to train the ResNet18 model. For details of the training process, see Supplementary Data S1.

#### Orientation ANN selection and evaluation

The ResNet18 model with square patch input was selected from Methods A to E (Supplementary Table S5), whose results were manually validated using a set of 80 structurally diverse grana. The tested methods included various CNNs, classical algorithmic methods using the Radon Transform, and combinations of ML and classical methods. We validated this 2-step orientation determination approach using a diverse set of 148 Arabidopsis, runner bean, pea, and tomato grana. The selected method correctly identified orientation in 95% of cases. In the remaining 3.65%, it was unable to estimate orientation, while in 1.35%, the estimated orientation was erroneous. These erroneous cases can be easily filtered by the user in the final HI stage of analysis due to the possibility of exporting the rotation image of each individual grana.

#### Period ANN

**Figure 6.**
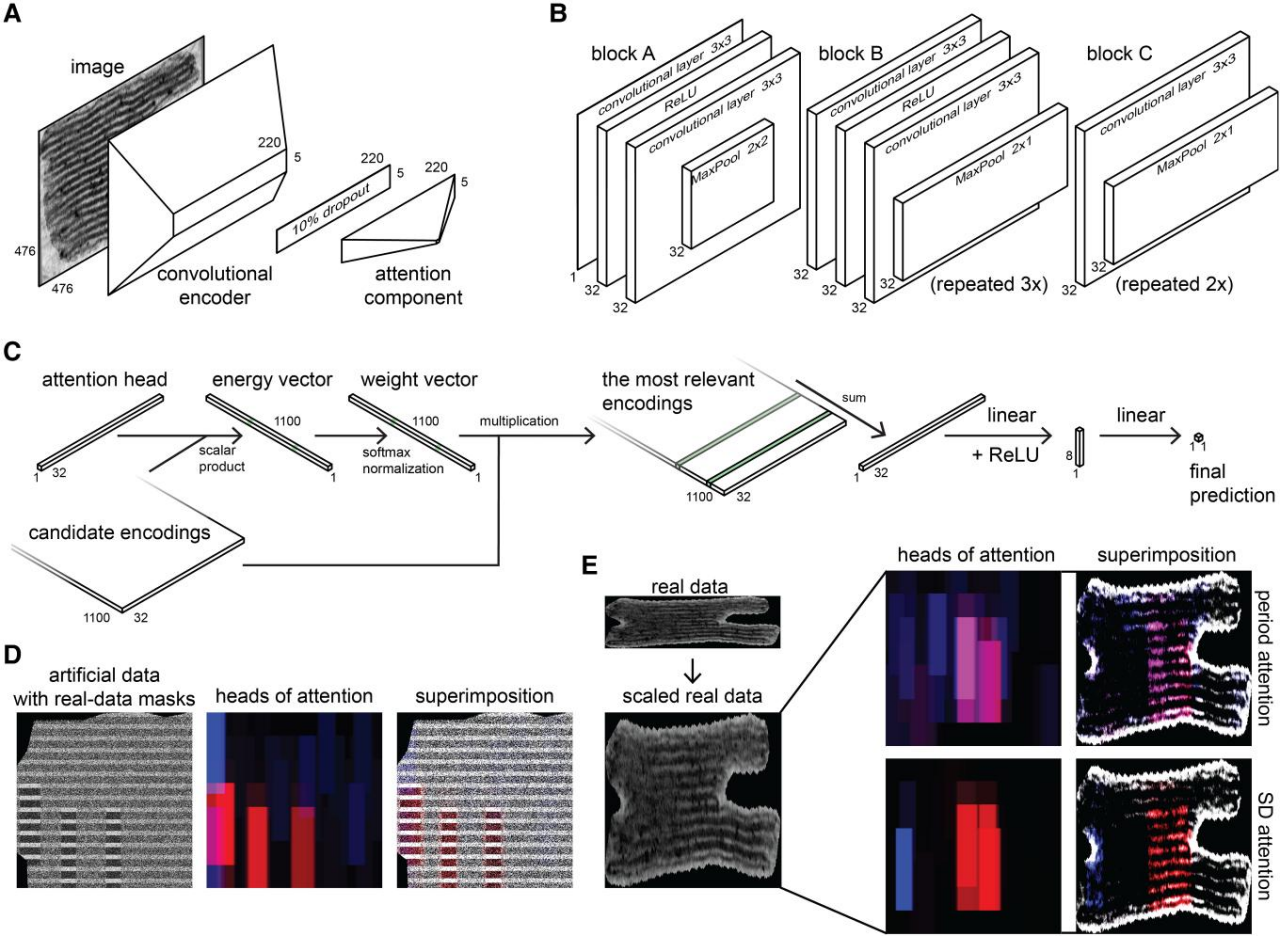
Period ANN architecture and operating principles of the attention component. **A** to **C)** Architecture of the components of period ANN with a showing the general ANN architecture. **B)** Presenting the architecture of Convolutional Encoder, additionally highlighting the points where the image dimensions are reduced (roughly) by half. **C)** Presenting the operations of attention component block D (for a single head and for 1 block D for simplicity). **D** and **E)** Illustration of period and Sd attention weights; left: input data; center: attention weights of output neurons visualized by showing the saturation of the corresponding receptive fields within the input image; right: superimposition of input image and the attention weights. **D)** Artificial data with real-data masks: in the image on the right the red attention head selects correctly the region of the highest period visibility. **E)** Real data presenting an example of nonuniform visibility of the period throughout the granum stack, with the region with the best period visibility detected by the red attention head. The pink rectangles in the image result from the overlap of the red and blue attention heads. Additionally, in the bottom row, the weights of the Sd attention component are shown.

Period ANN estimates both the SRD and the confidence (Sd of a predicted period distribution) from oriented granum image. Our custom-designed ANN for period estimation consists of a few larger building elements: the first one is the convolutional encoder, which is able to focus on overlapping fragments of the input image and encode those fragments using a 32-channel representation. Next, a dropout layer (Hinton et al. 2012), which randomly sets 10% of its input to zero during training, is applied to regularize the output of the convolutional encoder. Finally, the attention component of the network interprets the regularized representations through its 2 orthogonal attention heads and generates the final prediction, which is subsequently transformed using a nonlinear function. The neural network initially outputs a single prediction (the SRD value) and is trained using MSE loss. At a certain point during training, once the network has learned to predict the period value with sufficient accuracy, the model begins predicting the Sd of the underlying distribution, and the loss function transitions to Gaussian negative log likelihood loss. The details of this process and the associated mathematics are provided in Supplementary Data S2 and S4. The neural network architecture is presented in Fig. 6, A to C, and detailed below.

##### Convolutional encoder

The role of this component is to encode fragments of the input image (each such fragment corresponds to a receptive field of the output neuron). Specifically, we use 32-dimensional vectors for the encoding. The convolutional encoder consists of a building block A followed by 3 building blocks B followed by 2 building blocks C. Building block A has 1 input channel and 32 output channels. All intermediate channel counts are set to 32. It consists of a convolutional layer with 3-by-3 kernels followed by a ReLU nonlinearity followed by another convolutional layer with 3-by-3 kernels followed by a MaxPool layer with a 2-by-2 kernel and a 2-by-2 stride. Block A reduces the height and the width of its input image roughly twice. Building block B has all involved channel counts (input, output, and intermediate) equal to 32. It consists of a convolutional layer with 3-by-3 kernels followed by a ReLU nonlinearity followed by another convolutional layer with 3-by-3 kernels followed by a MaxPool layer with a 2-by-1 kernel and a 2-by-1 stride. Building block C also has all involved channel counts (input and output) equal to 32. It consists of a convolutional layer with 3-by-3 kernels followed by a MaxPool layer with a 2-by-1 kernel and a 2-by-1 stride. Blocks B and C reduce the height of their input image roughly twice, with the width (roughly) unchanged. As a result of these asymmetric architecture choices, each output neuron of the Convolutional Encoder has a receptive field of 220 by 38 neurons (height by width) with a stride of the receptive field of 64 by 2. Effectively, it thus encodes a 220 by 38 input image fragment into a 32-dimensional output encoding. Since the input image is 476-by-476 pixels and the output shape is 5 by 220 neurons, there are 1,100 overlapping fragments in an input image, each encoded into 32 output channels.

##### Attention component

The function of this component is to discern the most relevant encodings for the network's final prediction and to construct that prediction accordingly (Bahdanau et al. 2016). The attention component consists of 1 or 2 blocks D. They are used on the output of the dropout layer. A single building block D is utilized when predicting only the period length, whereas 2 blocks D are employed when predicting both the mean period value and its Sd. In this case, the 2 blocks operate in parallel, sharing the same input from the convolutional encoder through the dropout layer. Building block D consists of 2 learnable 32-parameter vectors called attention heads. It should be noted that an auxiliary loss component (see Equation (3) in Supplementary Data S3) ensures the orthogonality of the heads based on their mutual scalar product. The scalar products between an attention head and the 1,100 encodings of the overlapping fragments are computed to produce the energy vector of the encodings. The energy vector is subsequently normalized using the softmax nonlinearity. The resulting normalized energy vector serves as the weight vector for the encodings, with non-negative weights that sum to 1.0. The weights are then used to select the most relevant encodings for the network's prediction, resulting in a weighted sum of the encodings, which forms a 32-dimensional vector for a single attention head. Higher energy transforms to higher weights; it ensures that in the weighted sum the encodings with the highest energy dominate. The near orthogonality of the heads ensures they remain as independent as possible, allowing them to evaluate different aspects of the encodings as important or unimportant. Finally, the outputs of the 2 heads are concatenated, forming a 64-dimensional vector that passes through the 2 final layers of building block D. These layers are both linear, separated by a ReLU nonlinearity: the first layer reduces the dimensionality from 64 to 8, followed by the second layer, which reduces it further from 8 to 1. This resulting scalar represents the output of the attention component block D.

Figure 6B explains this process for a single head in 1 block D, and for this chart, only half of the first linear layer is presented with dimensions 32 by 8.

##### Final nonlinearity

The final nonlinearity used in the neural network is the reciprocal function (namely, f(x)=(h/x) with h=220, i.e. *h* is the receptive field height) in the case of the mean period prediction and the exponential function g(x)=exp(x) in the case of the Sd prediction. The rationale for selecting the nonlinear functions is as follows: the Sd must be strictly positive, which is why the exponential function was chosen; additionally, since the period length and the number of color changes (membranes) within the portion of the image encoded in the 32-channel representation are inversely related, the reciprocal function was used.

#### Period ANN training

The training of an ANN dedicated to period estimation consisted of multiple stages, including training using (i) artificial data, (ii) noisy artificial data with unaltered regions (attention training), (iii) artificial data with real-data masks, (iv) real data, (v) real data with the prediction of Sd, and (vi) real data with prediction of Sd on the full training set (Supplementary Fig. S4). In summary, for the training process, artificial datasets were generated randomly in an online manner, while for the real data, we used a set of 339 grana from various species, representing different grana shapes (Supplementary Table S2). The details of the training process are described in Supplementary Data S2.

#### Period ANN selection and evaluation

The method was evaluated on a separate test set initially consisting of 53 grana considered by 4 human experts as difficult (with the SRD boundaries hard to distinguish at times even by human experts). They were independently annotated in terms of the SRD by 4 human experts. Out of those 53 grana, only 40 had at least 2 independent annotations by human operators (the rest had zero or one annotation only, due to the difficulty of this set) and that selected 40 grana were further used as a test set. Out of the 7 model candidates (see Supplementary Data S2 for details on those 7 models), we selected the best network, the one that had the highest number of correct predictions: this network in 35 cases predicted the SRD value we considered correct, i.e. the value between the minimal and maximal value of those attributed by the humans, ±1 nm, with the mean predicted Sd of 1.09 nm. In the remaining 5 cases (considered wrong predictions), it had the mean predicted Sd of 3.46 nm (the predicted Sd was 2.19 nm or above in those 5 cases). These results demonstrate that the selected model can predict the SRD as accurately as humans can measure it from the same images. Furthermore, when the network is unable to make a reliable period prediction, it indicates this by outputting a higher than usual Sd. Thus, in our method, period predictions with Sd exceeding 2.5 are considered unreliable and are thus automatically rejected, preventing them from being presented to the user. The other 6 model candidates that were discarded at this stage had the number of wrong predictions between 6 and 11.

#### Attention component operating principle illustration

In Fig. 6B, we present the architecture, and in Fig. 6, D and E, we visualize the operating principle of the Attention Component of the period ANN.

In the TEM micrographs, within a single granum, there may be areas where thylakoids (layers) are clearly visible, but there may also be regions from which no meaningful information can be discerned, even by a human expert. Therefore, artificial datasets with various visibility of layers were prepared and used during the training process (Supplementary Fig. S4). A component of attention was integrated into the architecture of the period ANN, enabling it to identify the most valuable and informative sections of the granum from which the period can be determined.

The attention component works with the outputs of the convolutional encoder; note that each output neuron of the convolutional encoder encodes in its output channels information present in 1 vertical rectangle within an input image corresponding to the receptive field of this output neuron. Attention component uses its heads to compute weights for those encodings and (indirectly) for the rectangles within the input image. Those weights are depicted in Fig. 6, D and E by saturating the rectangles with red (for the first attention head) and blue (for the second attention head) in proportion to the corresponding weight. The pink rectangles within the image result from the superimposition of red and blue.

In the presented image corresponding to artificial data with real-data masks in Fig. 6D, the first (red) head learned to select the rectangles with the best quality within the image, while the second (blue) head seems to be selecting regions outside of the granum. This behavior of the red head is consistent throughout the data (it is not shown in Fig. 6), but the behavior of the blue head is less stable (not shown, either), and thus its role is hard to determine. It is important to emphasize that the network was not explicitly trained to prioritize high-quality regions of the image. Rather, the specialization of the red attention head emerged as an indirect consequence of penalization (via the loss function) for inaccuracies in estimating the period value.

For the real-data example, the first (red) head remains specialized on identifying regions with the highest quality and most informative content within the image, while the second (blue) head also shows a distinct specialization, though its exact function remains difficult to define (Fig. 6E). It indicates fragments of the input image, which contribute to the final prediction, though the underlying mechanism remains unclear.

Such approach achieves a high success rate of period prediction on real data, further validated by the Sd attention component. The Sd attention component was trained together with the period attention component, starting at Stage v. Its role is to identify grana regions where the period can be reliably detected. Thus, its red head retains the ability to select the regions with the best quality within the image. The blue head, while also specialized, functions differently than in the period attention component; it identifies parts of the input image relevant to the final Sd prediction, though the mechanism for this contribution is not fully understood.

Note that period ANN can predict the SRD value for grana stacks with thylakoid layers visible only partially, which is not possible for the human operator following the measuring protocol presented in Fig. 1G. In this case, the predicted Sd value can serve as an indicator for the user of the neural network's confidence in its period prediction.

### Accession numbers

GenBank/EMBL accession numbers are: AT1G44446 (CH1), AT4G01150 (CURT1A), AT2G46820 (CURT1B), AT1G52220 (CURT1C), AT4G38100 (CURT1D), AT5G67030 (ABA1), AT3G10230 (SZL1), AT1G08550 (NPQ1), AT3G11670 (DGD1), AT5G57030 (LUT2), AT1G31800 (LUT5), AT1G68830 (STN7), AT4G27800 (TAP38), AT1G77300 (CCR1), AT5G58770 (CPT7).

## Supplementary Material

kiaf212_Supplementary_Data

## Data Availability

Raw TEM data used for results in the manuscript are available at https://doi.org/10.58132/HTWCC1. The code is available on GitHub repository at github.com/center4ml/GRANA.

## Supplementary data

The following materials are available in the online version of this article.

**Supplementary Figure S1.** Representation of GRANA GUI.

**Supplementary Figure S2.** Best practices of microscopy imaging for efficient and reliable analysis using GRANA tool.

**Supplementary Figure S3.** Graphical illustration of the formula for calculating IoU and its application to granum segmentation.

**Supplementary Figure S4.** Exemplary data used in the process of training period ANN in subsequent stages of training.

**Supplementary Table S1.** Growth conditions of land plants used for TEM analysis.

**Supplementary Table S2.** Dataset details.

**Supplementary Table S3.** Characteristics of the 6 pretrained deep learning models used to select the final shape estimator.

**Supplementary Table S4.** Results of precision, recall, and F1 score metrics for 6 models tested in estimating grana shape.

**Supplementary Table S5.** Tested methods for granum mask orientation recognition.

**Supplementary Data S1.** Details of training of the orientation ANN.

**Supplementary Data S2.** Details of training of the period ANN.

**Supplementary Data S3.** Key metrics used during the evaluation of the shape ANN.

**Supplementary Data S4.** Loss functions.

## Dive Curated Terms

The following phenotypic, genotypic, and functional terms are of significance to the work described in this paper:

CH1 Gramene: AT1G44446
CH1 Araport: AT1G44446

CURT1A Gramene: AT4G01150
CURT1A Araport: AT4G01150

CURT1B Gramene: AT2G46820
CURT1B Araport: AT2G46820

CURT1C Gramene: AT1G52220
CURT1C Araport: AT1G52220

CURT1D Gramene: AT4G38100
CURT1D Araport: AT4G38100

ABA1 Gramene: AT5G67030
ABA1 Araport: AT5G67030

SZL1 Gramene: AT3G10230
SZL1 Araport: AT3G10230

NPQ1 Gramene: AT1G08550
NPQ1 Araport: AT1G08550

DGD1 Gramene: AT3G11670
DGD1 Araport: AT3G11670

LUT2 Gramene: AT5G57030
LUT2 Araport: AT5G57030

LUT5 Gramene: AT1G31800
LUT5 Araport: AT1G31800

STN7 Gramene: AT1G68830
STN7 Araport: AT1G68830

TAP38 Gramene: AT4G27800
TAP38 Araport: AT4G27800

CCR1 Gramene: AT1G77300
CCR1 Araport: AT1G77300

CPT7 Gramene: AT5G58770
CPT7 Araport: AT5G58770
