# Supplementary material for: GRANA: An AI–based tool for accelerating chloroplast grana nanomorphology analysis using hybrid intelligence
Source: Plant Physiol. 2025 May 23;198(2):kiaf212. doi: 10.1093/plphys/kiaf212 (PMC12142465; doi:10.1093/plphys/kiaf212)
Supplement: kiaf212_Supplementary_Data [file kiaf212_supplementary_data.pdf]

GRANA: An AI-based Tool for Accelerating Chloroplast Grana Nanomorphology Analysis Using Hybrid Intelligence

Alicja Bukat[1†], Marek Bukowicki[2,3†], Michał Bykowski[1], Karolina Kuczkowska[4], Szymon Nowakowski[2,5], Anna Śliwińska[2], Łucja Kowalewska[1*]

[1]Department of Plant Anatomy and Cytology, Faculty of Biology, University of Warsaw, Miecznikowa 1, 02-096, Warsaw, Poland.
[2]Center for Machine Learning, Faculty of Physics, University of Warsaw, Pasteura 5, 02-093, Warsaw, Poland.
[3]Centre of New Technologies, University of Warsaw, Banacha 2c, 02-093, Warsaw, Poland.
[4]Department of Ecology and Environmental Conservation, Faculty of Biology, University of Warsaw, Miecznikowa 1, 02-096, Warsaw, Poland.
[5]Institute of Applied Mathematics, Faculty of Mathematics, Informatics and Mechanics, University of Warsaw, Banacha 2, 02-097, Warsaw, Poland.

*Corresponding author(s). E-mail(s): lucja.kowalewska@uw.edu.pl;
†These authors contributed equally to this work.

Short title: AI-based tool for Automatic Grana Analysis

The author responsible for distribution of materials integral to the findings presented in this article in accordance with the policy described in the Instructions for Authors (https://academic.oup.com/plphys/pages/General-Instructions) is Łucja Kowalewska (lucja.kowalewska@uw.edu.pl).

Keywords: *electron microscopy, image analysis, artificial neural networks, chloroplast*

1 Supplementary Figures

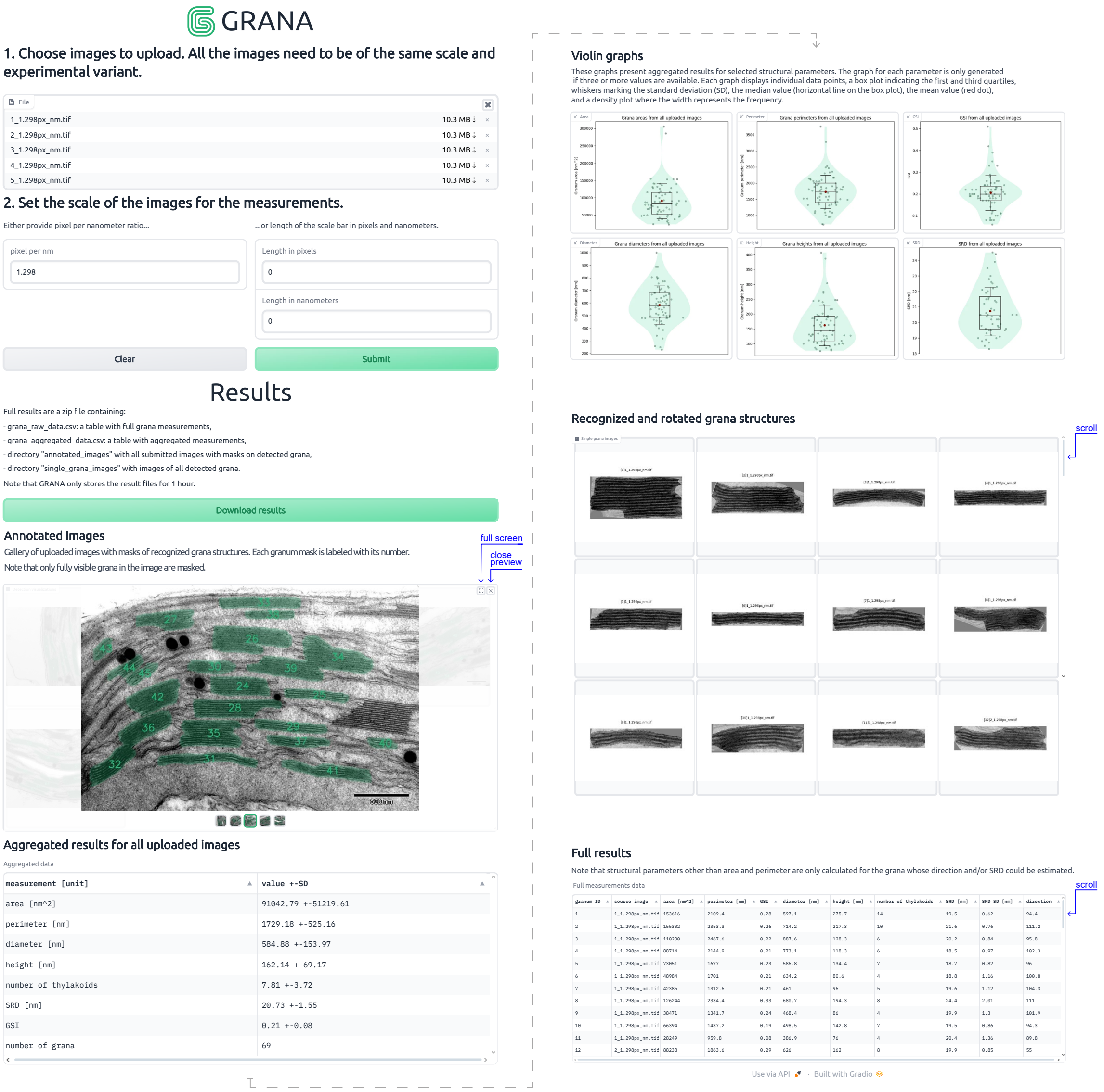

Supplementary Figure S1. Representation of GRANA graphical user interface (GUI). Input section of the GUI includes a data upload window (1) at the top and a scale setting area (2) below. The scale setting module consists of three text boxes. Users can either provide the pixels per nanometer ratio (on the left) or the length of the scale bar in pixels and nanometers (on the right) to have a ratio calculated automatically. After clicking the "Submit" button and allowing time for the analysis to complete, the output section displaying the results appears. By clicking the "Download results" button, results listed above the button can be downloaded. Results sections consist of five output components: i) a gallery of all uploaded TEM images with masks of recognized grana structures, ii) a table with aggregated results for all uploaded images, iii) violin graphs illustrating aggregated values for selected parameters, iv) a contact sheet of recognized and rotated grana masks and v) a table containing all raw data for each granum. Note that blue arrows with brief text clues are absent from the interface. TEM images presented in the figure are available as a sample dataset at chloroplast.pl/GRANA. Each violin graph presents individual data points, a box plot delineating the first and third quartiles, whiskers indicating the standard deviation (SD), a horizontal line on the box plot denoting the mean value, a square representing the median value, and a density plot where the width corresponds to the frequency.

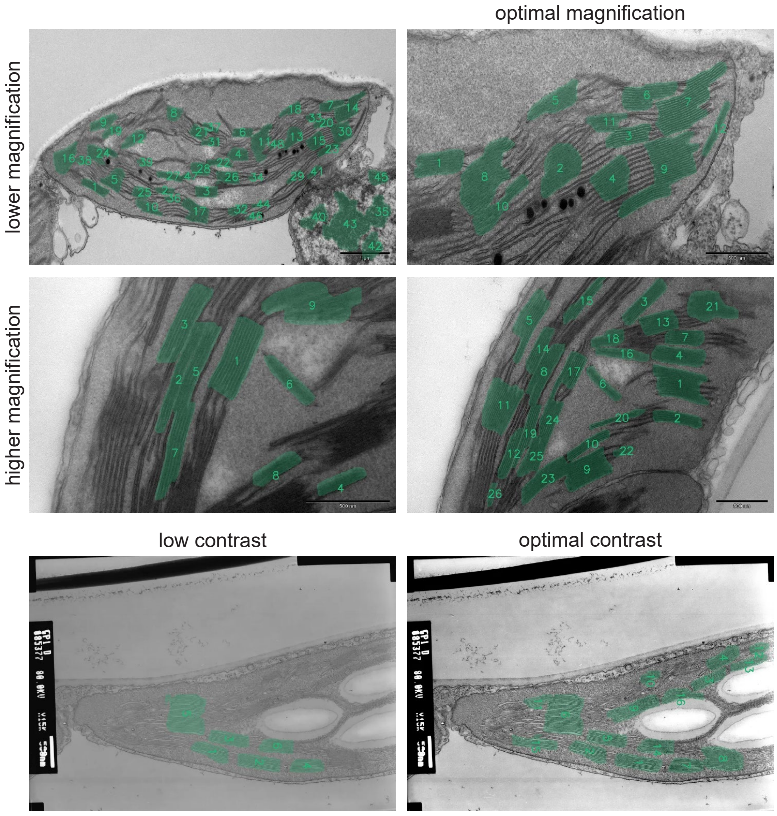

Best Practices of Microscopy Imaging for GRANA Analysis

Using too low magnification increases the risk of detecting other cellular structures resembling grana.

Low magnification is beneficial for quickly counting the number of grana visible in a chloroplast cross-section.

At low magnifications, GRANA may struggle to determine the SRD parameter due to insufficient image resolution at the nanometer scale.

Excessive magnification results in many edge-bound grana, significantly reducing the number of usable data points per image.

Insufficient image contrast can impair the detection of all visible grana.

Contrast can be improved post-capture, but it's crucial to ensure high-quality data acquisition during imaging.

Supplementary Figure S2. Best practices of microscopy imaging for efficient and reliable analysis using GRANA tool. Micrograph pairs illustrate non-optimal (left column) and optimal (right column) data for GRANA analysis. We recommend adhering to the following practices: when selecting TEM images, consider both quality and magnification. Ideal micrographs are well-contrasted with distinctly visible grana. Avoid images showing entire chloroplasts in one frame, as grana may be too small for accurate structural parameter estimation due to poor visibility of layers and lumen fraction. All scale bars represent 500 nm, except for the top image in the left column, where the scale bar represents 1 μm.

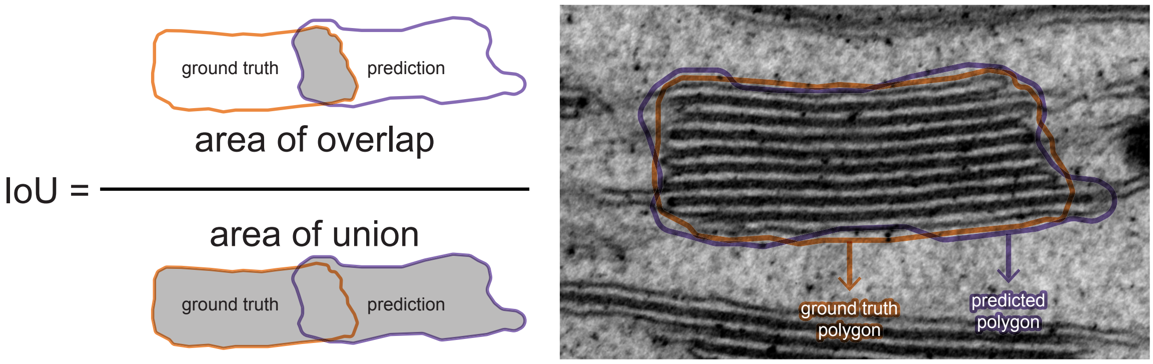

Supplementary Figure S3. Graphical illustration of the formula for calculating Intersection over Union (IoU) and its application to granum segmentation. On the left: the diagram explaining the IoU formula, which quantifies segmentation accuracy as the ratio of the area of overlap to the area of union between the ground truth (orange) and predicted (purple) polygon masks. On the right: the TEM image of a granum stack showing examples of ground truth and predicted polygonal annotations used to compute IoU.

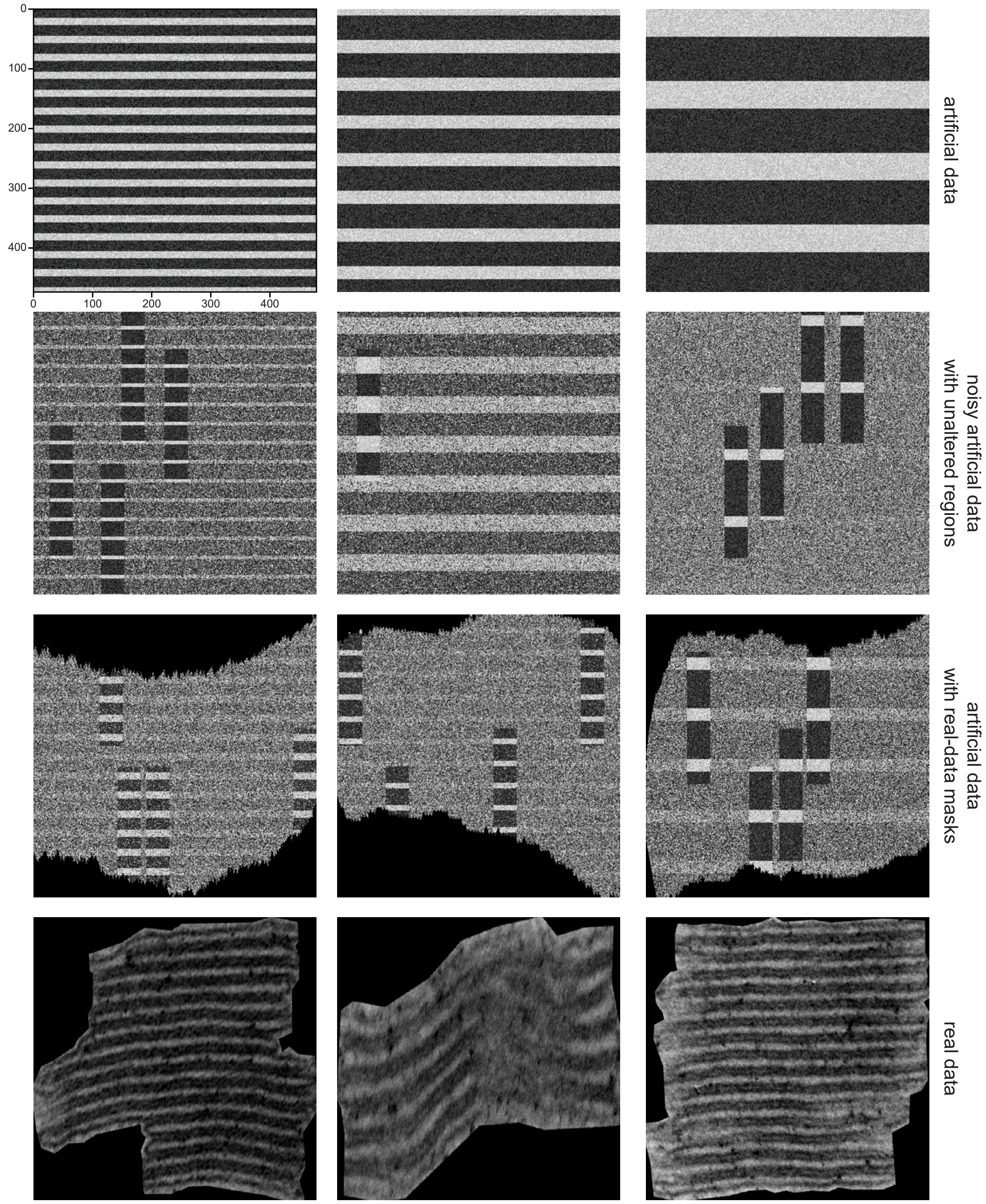

Supplementary Figure S4. Exemplary data used in the process of training period ANN in subsequent stages of training. For each data set (artificial, noisy artificial data with unaltered regions, artificial data with real-data masks, and real data) three representative examples used during training are presented. Note that for the artificial data, the ground truth period was known, as these data were generated synthetically, while for the real data, the ground truth period was determined through manual measurements.

2 Supplementary Tables

Supplementary Table S1. Growth conditions of land plants used for TEM analysis.

experimental variants	species	time of growth	place of growth	photoperiod	light intensity
Arabidopsis *chl1-1* mutant	*Arabidopsis thaliana* (L.) Heynh.	5 weeks	growth chamber	16 h day / 8 h night	120 μmol photons $m^{-2} s^{-1}$
Arabidopsis *curt1abcd* mutant	*Arabidopsis thaliana* (L.) Heynh.	4 weeks	growth chamber	12 h day / 12 h night	120 μmol photons $m^{-2} s^{-1}$
Arabidopsis *aba1-6* mutant	*Arabidopsis thaliana* (L.) Heynh.	8 weeks	growth chamber	8 h day / 16 h night	70 μmol photons $m^{-2} s^{-1}$
Arabidopsis *szl1-1 npq1-2* mutant	*Arabidopsis thaliana* (L.) Heynh.	8 weeks	growth chamber	8 h day / 16 h night	70 μmol photons $m^{-2} s^{-1}$
Arabidopsis *dgd1* mutant	*Arabidopsis thaliana* (L.) Heynh.	8 weeks	growth chamber	8 h day / 16 h night	120 μmol photons $m^{-2} s^{-1}$
Arabidopsis *lut2-1* mutant	*Arabidopsis thaliana* (L.) Heynh.	8 weeks	growth chamber	8 h day / 16 h night	70 μmol photons $m^{-2} s^{-1}$
Arabidopsis *lut5-1* mutant	*Arabidopsis thaliana* (L.) Heynh.	8 weeks	growth chamber	8 h day / 16 h night	70 μmol photons $m^{-2} s^{-1}$
Arabidopsis *lut5-1* extensive light mutant	*Arabidopsis thaliana* (L.) Heynh.	7 weeks<br>1 week	growth chamber	8 h day / 16 h night	70 μmol photons $m^{-2} s^{-1}$,<br>120 μmol photons $m^{-2} s^{-1}$
Arabidopsis *stn7-8* mutant	*Arabidopsis thaliana* (L.) Heynh.	5 weeks	growth chamber	16 h day / 8 h night	60 μmol photons $m^{-2} s^{-1}$
Arabidopsis *tap38* mutant	*Arabidopsis thaliana* (L.) Heynh.	5 weeks	growth chamber	16 h day / 8 h night	60 μmol photons $m^{-2} s^{-1}$
Arabidopsis *ccr1-1* mutant	*Arabidopsis thaliana* (L.) Heynh.	8 weeks	growth chamber	8 h day / 16 h night	70 μmol photons $m^{-2} s^{-1}$
Arabidopsis growth chamber	*Arabidopsis thaliana* (L.) Heynh.	5 weeks	growth chamber	16 h day / 8 h night	120 μmol photons $m^{-2} s^{-1}$
Arabidopsis growth chamber low light	*Arabidopsis thaliana* (L.) Heynh.	5 weeks	growth chamber	16 h day / 8 h night	60 μmol photons $m^{-2} s^{-1}$
Young Arabidopsis growth chamber low light	*Arabidopsis thaliana* (L.) Heynh.	5 days<br>3 days	growth chamber	 16 h day / 8 h night	etiolation<br>60 μmol photons $m^{-2} s^{-1}$
Arabidopsis growth chamber 9 hour day	*Arabidopsis thaliana* (L.) Heynh.	4 weeks	growth chamber	9 h day / 15 h night	150 μmol photons $m^{-2} s^{-1}$
Arabidopsis greenhouse	*Arabidopsis thaliana* (L.) Heynh.	4 weeks	greenhouse	natural photoperiod	natural light
Arabidopsis greenhouse heat stress	*Arabidopsis thaliana* (L.) Heynh.	4 weeks	greenhouse	natural photoperiod	natural light
Arabidopsis *cpt7* mutant greenhouse	*Arabidopsis thaliana* (L.) Heynh.	4 weeks	greenhouse	natural photoperiod	natural light
Young runner bean	*Phaseolus coccineus* L.	6 days<br>3 days	growth chamber	 16 h day / 8 h night	etiolation<br>120 μmol photons $m^{-2} s^{-1}$
Runner bean	*Phaseolus coccineus* L.	10 days	growth chamber	16 h day / 8 h night	120 μmol photons $m^{-2} s^{-1}$
Tomato	*Solanum lycopersicum* L.	3 weeks	growth chamber	16 h day / 8 h night	120 μmol photons $m^{-2} s^{-1}$
Pea	*Pisum sativum* L.	2 weeks	growth chamber	16 h day / 8 h night	120 μmol photons $m^{-2} s^{-1}$
Rubber tree	*Ficus elastica* Roxb. ex Hornem. 1819	mature plant<br>+ 2 weeks of adaptation<br>to laboratory conditions	greenhouse	natural photoperiod	natural light
White mustard	*Sinapis alba* L.	3 weeks	growth chamber	16 h day / 8 h night	120 μmol photons $m^{-2} s^{-1}$
Cultivated tobacco	*Nicotiana tabacum* L.	4 weeks	growth chamber	12 h day / 12 h night	120 μmol photons $m^{-2} s^{-1}$
Rose mallow	*Hibiscus* × *rosa-sinensis* L.	mature plant<br>+ 2 weeks of adaptation<br>to laboratory conditions	greenhouse	natural photoperiod	natural light
Spider plant	*Chlorophytum comosum* (Thunb.) Jacques	mature plant<br>+ 2 weeks of adaptation<br>to laboratory conditions	greenhouse	natural photoperiod	natural light

Supplementary Table S2. Dataset details.

Training set for grana detection	grana	species
A	380	Arabidopsis (growth chamber, growth chamber low light, greenhouse, greenhouse heat stress, *dgd1*)
B	618	Arabidopsis (young growth chamber low light, *curt1abcd*, *aba1-6*, *dgd1*, *lut2-1*, *lut5-1*, *ccr1-1*), Rubber tree, Rose mallow, Spider plant
C	1039	Runner bean, Pea
D	1164	Arabidopsis (growth chamber, greenhouse, greenhouse heat stress, *stn7-8*, *tap38*, *cpt7*)
E	2439	Arabidopsis (growth chamber 9 hour day), Runner bean (photographic films), Pea (photographic films)

Training set for measurements	grana	species
Test set	53	Arabidopsis (greenhouse heat stress)
Period estimation	339	Arabidopsis (growth chamber 9 hour day, greenhouse heat stress, *szl1-1 npq1-2*, *lut5-1* extensive light)
Orientation estimation	135	Arabidopsis (growth chamber, growth chamber low light, greenhouse, greenhouse heat stress, *dgd1*), Runner bean

Training sets used for analysis presented in the results section	grana	species
Figure 3	183	Cultivated tobacco
Figure 4	1378	Arabidopsis (growth chamber low light, greenhouse, greenhouse heat stress, *chl1-1*), Young runner bean, Runner bean, Tomato, Pea, Rubber tree

Supplementary Table S3. Characteristics of the 6 pre-trained deep learning models used to select the final shape estimator.

model ID	model type	stages of estimation	parameters	image scaling
Model_A	Mask R-CNN[1]	2	default (horizontal flip only)	no
Model_B	YOLOv8s[2]	1	default	yes
Model_C	YOLOv8s	1	modification of perspective, degree, shear	no
Model_D	YOLOv8s	1	modification of perspective, degree, shear	yes
Model_E	Mask R-CNN	2	brightness, contrast, horizontal and vertical flips, cutout[3]	no
Model_F	YOLOv8s	1	modification of intersection over union	yes

[1]He K, Gkioxari G, Dollár P, Girshick R. 2017. Mask R-CNN. arXiv. 2017; arXiv:1703.06870v3. doi:10.48550/arXiv.1703.06870, preprint: not peer reviewed.

[2]Jocher G, Qiu J, Chaurasia A. 2023. Ultralytics YOLO. (Version 8.0.0) [software]. Ultralytics; 2023. Available at: https://github.com/ultralytics/ultralytics. Accessed May 26, 2025.

[3]DeVries T, Taylor GW. 2017. Improved regularization of convolutional neural networks with cutout. arXiv. 2017; arXiv:1708.04552v2. doi:10.48550/arXiv.1708.04552, preprint: not peer reviewed.

Supplementary Table S4. Results of precision, recall, and F1 score metrics for 6 models tested in estimating grana shape. Metrics were calculated across 6 Intersection over Union (IoU) thresholds: 0.4, 0.5, 0.6, 0.7, 0.8, and 0.9. The highest values are bolded.

PRECISION

IoU threshold	model_A	model_B	model_C	model_D	model_E	model_F
0.4	0.93	0.89	0.72	**0.94**	0.90	**0.94**
0.5	0.90	0.88	0.70	0.93	0.88	**0.94**
0.6	0.84	0.86	0.65	**0.91**	0.81	0.90
0.7	0.78	0.82	0.60	**0.89**	0.74	0.86
0.8	0.70	0.72	0.45	**0.83**	0.65	0.77
0.9	0.31	0.38	0.13	**0.39**	0.31	0.34

RECALL

IoU threshold	model_A	model_B	model_C	model_D	model_E	model_F
0.4	0.67	0.86	0.82	**0.90**	0.73	0.86
0.5	0.65	0.85	0.80	**0.89**	0.71	0.86
0.6	0.62	0.83	0.73	**0.87**	0.65	0.83
0.8	0.50	0.70	0.51	**0.79**	0.53	0.71
0.9	0.23	**0.38**	0.17	**0.38**	0.25	0.31

F1 SCORE

IoU threshold	model_A	model_B	model_C	model_D	model_E	model_F
0.4	0.78	0.88	0.77	**0.92**	0.80	0.90
0.5	0.76	0.87	0.75	**0.91**	0.79	0.90
0.6	0.71	0.84	0.69	**0.89**	0.72	0.86
0.7	0.66	0.81	0.63	**0.87**	0.67	0.83
0.8	0.59	0.71	0.48	**0.81**	0.58	0.74
0.9	0.26	0.38	0.15	**0.38**	0.28	0.32

Supplementary Table S5. Tested methods for granum mask orientation recognition.

method ID	patched input	Radon transform	ANN
A	-	+	-
B	-	+	-
C	-	-	ResNet18
D	(with circle mask)	-	ResNet18
E	(with circle mask)	+	ResNet18

3 Supplementary Data

3.1 Supplementary Data S1 – Details of training of the orientation ANN

3.1.1 Data

We used a dataset of approximately 122 000 grana patch images. For training, we randomly split this dataset into training and test sets, with 90% of the samples in the training set and 10% in the validation set, used to select the optimal model and the training time. Data normalization based on defined mean and SD was applied to each image to enhance model performance and stability.

3.1.2 CNN Training

Augmentations

We applied Gaussian blur to images in both training and testing phases. In training, blur intensity varied within defined limits, while in testing, it remained constant. Training images were also randomly rotated and flipped.

Backbone selection

We experimented with different convolutional backbones using pytorch-image-models package implementation, looking for a fast and efficient solution. After examining `resnet18`, `mobilenetv3_small_050` and `lcnet_050`, we decided to use `resnet18`, which performs equally well and is a lasting standard in computer vision.

Criterion

Due to the orientation ambiguity (rotations ϕ are equivalent to $\phi + \pi$), we could not use the MSE loss (see equation (5) in 3.4 Supplementary Data S4) which would treat angles differing by π radians as significantly different. Instead, we used the Cosine Similarity loss (see equation (11) in 3.4 Supplementary Data S4).

3.1.3 Downstream Pipeline

The preliminary orientation estimate is the circular mean of estimates made on patches. We experimented with a different number of granum patches used at inference and set it to 32. Larger number of patches (64, 128) did not lead to noticeable better performance.

The confidence of orientation estimate is derived from the circular SD of predictions for the patches. The threshold, above which the orientation is labeled as unknown, was manually adjusted on a test set.

After the orientation ANN training we discovered that its output does not always match human expectations. In particular, the orientation of curved and partially blurred grana may be ambiguous, and its accurate orientation estimation requires taking into account its shape. To this end we proposed an orientation correction method which may adjust the ANN orientation estimate in the range [-10,10] degrees range. The orientation correction minimizes the rectangular envelope of a granum. Such an approach cannot be applied on raw data (as ANN replacement) because it often gives erroneous results, e.g. for tall grana. However, the envelope minimization is successful in correcting ANN errors. This additional step was validated on a dataset of 50 grana.

3.2 Supplementary Data S2 – Details of training of the period ANN

Training of the period ANN consisted of several stages.

3.2.1 Artificial Data Training

The aim of this training stage was to initiate the network, focusing primarily on training the Convolutional Encoder using images significantly simpler than actual grana.

In this stage, the Attention Component of the network comprised just one building block D, restricting the network to predicting only the period length. Given the uniform quality of the input images, the Attention Component remained mostly inactive and transparent throughout this stage.

The training images were generated artificially. The period width was uniformly sampled from between 20 and 140, light fraction was sampled uniformly from 15 to 45 percent; a 476 by 476 image was then filled in by alternating light and dark stripes, with the light ones occupying the previously sampled fraction of the overall period. The intensity of each pixel in the dark stripes were sampled uniformly from between 0 and 100, while the intensity of each pixel in the light ones were sampled uniformly from between 156 and 255. The epoch length was the same as for the real dataset.

After the satisfactory validation prediction quality for this stage was achieved, the training was stopped after aprox. 200 epochs.

3.2.2 Attention Training

The objective of this training stage was to initiate the Attention Component, which, while present, remained effectively unused during the previous stage.

During this stage, the Attention Component in the network consisted of only one building block D, limiting the network to predicting only the period length.

The training images were generated artificially, as in the previous stage, with the added inclusion of normal noise. This noise was zero-centered, and its SD varied across input images, with the parameter uniformly sampled between 0.0 and 100.0 prior to image generation. To train the attention mechanism, between 1 and 4 rectangles (matching the receptive field size and position, potentially overlapping) were excluded from the noising process and retained unaltered information about the period length. This approach enabled the network to learn how to use the attention mechanism to select the most informative parts of the image.

The training steadily improved the validation error. We stopped the training after the satisfactory validation prediction quality for this stage was achieved, i.e. the training was stopped after aprox. 460 epochs.

3.2.3 Artificial Data with Real Masks

The objective of this training stage was to gradually increase the complexity of the data, approaching the difficulty of real grana. To achieve this, normal noise was added to the images generated as in the previous stage, with the noise's SD now uniformly sampled between 0.0 and 20.0, including the regions initially excluded from the noise to train the attention mechanism. Additionally, real masks were subsampled from the training set of real grana and applied to the artificially generated training images, with the area outside the mask set to zero. A similar procedure was applied to the artificial validation set using masks from the real validation set.

Finally, the training images were further subsampled, symmetrically transformed, tilted, and had random amounts of noise, resembling microscopic noise, added. These steps were implemented as part of the image augmentation process.

As in previous stages, the Attention Component of the network at this stage contained only a single building block D, resulting in the network predicting solely the period length.

We stopped the training after the satisfactory validation prediction quality for this stage was achieved, i.e. the training was stopped after aprox. 550 epochs.

3.2.4 Real Data

In this stage the network was trained on the real grana from the training set and validated on the real grana from the validation set.

As in previous stages, the Attention Component of the network at this stage contained only a single building block D, resulting in the network predicting solely the period length.

To enhance the diversity of the training data, the input images were subsampled, symmetrically transformed, tilted, and subjected to randomly generated noise resembling microscopic noise, as part of the image augmentation procedure.

We stopped the training after the satisfactory validation prediction quality for this stage was achieved, i.e. the training was stopped after aprox. 430 epochs.

3.2.5 Real Data with Prediction of Standard Deviation

In this stage the network was trained on the real grana from the training set and validated on the real grana from the validation set.

In contrast to previous stages, the Attention Component of the network in this stage consisted of two blocks D, enabling the network to predict not only the period length $\hat{y}$ (interpreted as the mean period value) but also its confidence in the prediction by estimating $\hat{\text{sd}}$, the SD of the underlying normal distribution. To build the network in this stage from the previous one, the second building block D was initially cloned from the first and subsequently trained independently. The transition loss L_3, shifting from the MSE-based loss to the GNLL-based loss, was calculated over the first 30 epochs with the formula (10), after which only the GNLL-based L_2 loss (9) was applied exclusively.

As before, to improve the diversity of the training data, the images were subsampled, symmetrically transformed, tilted, and infused with randomly generated noise mimicking microscopic noise, all as part of the image augmentation strategy.

We stopped the training after the satisfactory validation prediction quality for this stage was achieved and was not improving, i.e. the training was stopped after 1112 epochs.

3.2.6 Real Data with Prediction of Standard Deviation—Full Set

In this stage, the network was further trained using the real grana from both the training and validation sets combined, with no separate validation set used to monitor training progress.

In this stage, the Attention Component of the network consisted of two blocks D, allowing it to predict both the period length $\hat{y}$ (interpreted as the mean period value) and the confidence in its prediction by estimating $\hat{\text{sd}}$. As a result, the network exclusively utilized the GNLL-based L_2 loss (9).

As in previous stages, to enhance the diversity of the training data, the input images were subsampled, symmetrically transformed, tilted, and subjected to random noise simulating microscopic noise, as part of the image augmentation process.

We stopped the training when the network's training error, which we monitored throughout, began to increase—this occurred after approximately 600 epochs. Prior to this, we saved seven network candidates: four corresponding to local minima in the training error and three at predetermined training intervals, specifically after 0, 200, and 300 epochs. These seven network candidates were then evaluated using the method outlined in the main text, in the Materials and Methods section - *Period ANN selection and evaluation*.

3.3 Supplementary Data S3 – Key metrics used during the evaluation of the shape ANN

3.3.1 Scores of Confusion Matrix

1. True Positives (TP): The number of cases the model correctly predicted a positive outcome (the actual, ground truth outcome was positive).
2. True Negatives (TN): The number of cases the model correctly predicted a negative outcome (the actual, ground truth outcome was negative).
3. False Positives (FP): The number of cases the model incorrectly predicted a positive outcome (the actual, ground truth outcome was negative).
4. False Negatives (FN): The number of cases the model incorrectly predicted a negative outcome (the actual, ground truth outcome was positive).

3.3.2 Metrics

Precision

Precision is calculated by dividing the number of true positive results by the total number of positive results. It addresses how accurate the model's positive predictions are, measuring the amount of irrelevant results (with higher precision indicating less irrelevant output). Precision formula:

$$\text{Precision} = \frac{TP}{TP + FP} \tag{1}$$

where: TP+FP are the total predicted objects.

Recall

Recall, also known as sensitivity or true positive rate, is calculated by dividing the number of true positive results by the total number of samples that should have been classified as positive. Recall indicates how well the model identified all relevant samples. A higher recall indicates that fewer actual positive samples were missed. Recall formula:

$$\text{Recall} = \frac{TP}{TP + FN} \tag{2}$$

where TP+FN are the total ground truth objects.

F1 Score

The F1 score is a metric that balances precision and recall. It's calculated as the harmonic mean of the precision and recall scores. A high F1 score indicates high levels of both precision and recall. F1 score formula:

$$\text{F1 Score} = 2 \cdot \frac{\text{Precision} \cdot \text{Recall}}{\text{Precision} + \text{Recall}} = \frac{2 \cdot TP}{2 \cdot TP + FP + FN} \tag{3}$$

Intersection over Union (IoU)

IoU, also known as Jaccard Index, measures the overlap between the ground truth bounding boxes and the predicted bounding boxes (Redmon et al. 2016). Specifically, let B_p and bounding box and the ground truth bounding box, respectively. The Intersection over Union is a metric that measures the similarity between these two bounding boxes. It is defined as the ratio of the area of the intersection of the two bounding boxes to the area of their union:

$$\text{IoU}(B_p, B_g) = \frac{|B_p \cap B_g|}{|B_p \cup B_g|} \tag{4}$$

where |X| denotes an area of any planar set X. This is also illustrated in Supplementary Figure S3.

3.4 Supplementary Data S4 – Loss functions

Below we provide details on loss functions used during training of the artificial neural networks.

3.4.1 Mean Square Error

Mean Square Error (MSE) loss is a very typical choice of a loss function. It models the mean squared distance between the ground truth values y_i and the predictions $\hat{y}_i$, $i = 1, \ldots, n$.

$$\text{MSE}(y, \hat{y}) = \frac{\sum_{i=1}^{n}(y_i - \hat{y}_i)^2}{n}. \tag{5}$$

The MSE loss can also be derived from the negative log likelihood of the data y_i, $i = 1, \ldots, n$, under the assumption the data comes from the normal distribution centered at $\hat{y}_i$ with the covariance matrix proportional to the identity matrix (i.e. all its components having the same SD, say 1.0).

3.4.2 Gaussian Negative Log Likelihood

The negative log likelihood loss model allows us to go one step further: not only to predict the values (with the use of means of our distribution, i.e. $\hat{y}_i$), but also to predict *how certain we are that we are correct* with the use of $\hat{\text{sd}}_i$, the prediction of the SD. Namely, if we assume that y_i comes from the normal distribution centered at $\hat{y}_i$ with the SD $\hat{\text{sd}}_i$ (which may differ from case to case), the resulting negative log likelihood loss is the Gaussian Negative Log Likelihood (GNLL) loss:

$$\text{GNLL}(y, \hat{y}, \hat{\text{sd}}) = \frac{1}{n}\sum_{i=1}^{n}\left(\log(\hat{sd}_i) + \frac{(y_i - \hat{y}_i)^2}{2\hat{\text{sd}}_i}\right). \tag{6}$$

3.4.3 Period ANN Specific Loss Functions

To ensure that the network attention heads h_1 and h_2 are orthogonal, we introduce an additional loss component which measures the distance of the scalar product of h_1 and h_2 to 0:

$$\text{ORT}(h_1, h_2) = \text{MSE}(h1 \cdot h2,\ 0). \tag{7}$$

For training of the network predicting only the period value, we use the following loss function:

$$L_1(h1, h2, y, \hat{y}) = \text{MSE}(y, \hat{y}) + \lambda\ \text{ORT}(h_1, h_2). \tag{8}$$

For training of the network predicting the mean period value and its SD, we use the following loss function:

$$L_2(h1, h2, y, \hat{y}, \hat{\text{sd}}) = \text{GNLL}(y, \hat{y}, \hat{\text{sd}}) + \lambda\ \text{ORT}(h_1, h_2), \tag{9}$$

with the exception, that for the transition period over the first 30 epochs we use the following interpolating loss function (let $i = 0, \ldots, 29$ be the epoch number):

$$\begin{aligned} L_3(i, h_1, h_2, y, \hat{y}, \hat{\text{sd}}) = &\cos(\frac{i}{30}\frac{\pi}{2})\ \text{MSE}(y, \hat{y}) + \\ &\sin(\frac{i}{30}\frac{\pi}{2})\ \text{GNLL}(y, \hat{y}, \hat{\text{sd}}) + \\ &\lambda\ \text{ORT}(h_1, h_2). \end{aligned} \tag{10}$$

Above in (8), (9) and (10), λ is a weight and we set $\lambda = 0.1$ in all three cases.

3.4.4 Orientation ANN Specific Loss Function

During the orientation ANN training we used Cosine Similarity (CS) loss to penalize incorrect rotation predictions for grana. This loss function maximally penalizes a predicted angle $\hat{a}$ that differs by $\frac{\pi}{2}$ radians from the ground truth angle a, with the penalty decreasing as the prediction aligns

more closely or diverges beyond the $\frac{\pi}{2}$ threshold from the ground truth. Notably, angles differing by exactly π are treated as equivalent due to the top-down symmetry in the images:

$$\text{CS}(a, \hat{a}) = 1 - \cos\left(2(a - \hat{a})\right). \tag{11}$$
